# Investigation of the effectiveness of the thermal conductivity of the Bingham fluid subjected to the dispersion of nanoparticles and dissipation effects

**DOI:** 10.1038/s41598-026-60219-0

**Published:** 2026-07-22

**Authors:** Kamla Imtiaz, M. Nawaz, Sayer Obaid Alharbi, Saleh Mousa Alzahrani

**Affiliations:** 1 Department of Applied Mathematics and Statistics, Institute of Space Technology, P.O.BOX 2750, Islamabad, 44000 Pakistan; 2 Mathematics Department, College of Science Al-Zulfi, Majmaah University, Majmaah, 11952 Saudi Arabia; 3 Department of Mathematics, University College in Al-Qunfudhah, Umm Al- Qura University, Makkah, Saudi Arabia

**Keywords:** Viscoplastic fluid, Thermal effectiveness, Yield stress, Porous medium, Heat transfer rate, Bingham rheological model, Engineering, Mathematics and computing, Nanoscience and technology, Physics

## Abstract

Several fluids exhibit viscoplastic behavior, and several rheological models for viscoplastic fluids have been proposed. The Bingham-Papanastasiou rheological model is one of them. This article examines heat transfer in a Bingham fluid over a heated wedge. Three types of nanoparticles are considered to be dispersed in the Bingham fluid, and their impact on enhancing the thermal effectiveness of the Bingham fluid is examined. For this purpose, the basic equations of fluid dynamics, energy equations, and Bingham-Papanastasiou rheological models are used for modeling of heat transfer under magnetic and porous medium forces. A set of partial differential equations (PDEs) is transformed into ordinary differential equations (ODEs) and numerically solved under no-slip boundary conditions by applying the Galerkin finite element method (GFEM). The roles of magnetic field and porous media forces in thermal enhancement are noted, and it is found that both porous medium and magnetic forces are not favorable for enhancing thermal transfer. An increase in thermal radiation intensity leads to an increase in the Nusselt number. The porous medium causes dissipation due to the resistive force experienced by the fluid flow. For thermal and cooling systems to be thermally efficient and sustainable, the working Bingham fluid should not be heat dissipative, either because of Joule heating or viscous dissipation. Among the different types of nanofluids, the most prominent is the case of tri-nanofluids due to their higher thermal conductivity and stronger fluid–thermal interactions, which makes them more sensitive to parameter variations compared to mono- and di-nanofluids. This study predicts that the skin friction coefficient increases when the Bingham number is increased. Hence, the viscoplastic fluids with higher yield stress exert higher drag on the solid surface to which such fluid interacts. Moreover, the viscoplastic fluid (the Bingham fluid) exerts higher drag on the surface in comparison with Newtonian fluids.

## Introduction

 Heat transfer is a fundamental component of many natural and artificial processes, and heat transfer in fluids outlines everything from weather patterns to contemporary machinery. The climate system in nature is driven by the movement of heat through the oceans and atmospheric currents, which redistributes thermal energy globally and permits life to thrive in various settings. Heat transmission in fluids is the foundation of engineering technologies such as chemical processing, power production, refrigeration, automobiles, nuclear reactors, etc. It controls how engines transform thermal energy into mechanical energy, how heat exchangers maintain an efficient energy transfer, and how cooling systems keep anything from laptops to spacecraft from overheating. Since even small inefficiencies can influence environmental management, technological breakthroughs, and sustainable energy consumption, it is crucial to comprehend and optimize these processes. Heat transfer in fluid has been studied to unfold various significant aspects. Pandey et al.^1^ studied heat transfer in a non-Newtonian fluid in a square-type container with a circular obstacle. Kushwaha et al.^2^ theoretically analyzed heat transfer in a non-Newtonian fluid-filled tube-type heat exchanger. Dong et al.^3^ studied heat transfer in a fluid in a microchannel using an optimization technique. Kumar and Verma et al.^4^ discussed heat transfer in fluid associated with a sinusoidal protrusion rib in a solar air heater. Wang et al.^5^ examined mixing of non-Newtonian fluid in a Sulzer mixer reactor subjected to heat transfer. Shaw and Guerrero^6^ discussed the applications related to heat transfer in non-Newtonian fluids. Sirait et al.^7^ theoretically examined the process of heat transfer in a non-Newtonian fluid.

Today’s world is facing a great challenge, and numerous efforts have been put forward to address this challenge. One of the remarkable steps towards austerity measures is the efficient transport of available energy for its optimal use. The major means of transport of heat energy are fluids, which are frequently used as a medium for transporting heat in numerous engineering processes and mechanisms. The biggest challenge in this connection is that fluids have lower thermal conductivity in comparison with solids, due to which optimal transport of heat is not achieved. This flaw can be minimized via the augmentation of several arrangements. Passive and active approaches have been used. However, the latest one is to raise the thermal conductivity of the fluid. The best and most innovative method of increasing the thermal conductivity of the fluid is the dispersion of nanoparticles in the fluid. This method is extensively used nowadays, and extensive research has been published on this topic. Akbari et al.^8^ studied the combined effects of velocity and dimensions of nanoparticles on heat transfer in a non-Newtonian fluid. Zhuang et al.^9^ studied the importance of nanoparticles with respect to thermal enhancement. Mohamed et al.^10^ analyzed heat transfer enhancement because of the nanoparticles in a fluid passing over a heated sphere. Hakeem et al.^11^ studied the combined effects of nanoparticles on thermal enhancement in convective fluid flow subjected to uniform heat flux at the flat plate. Saleem et al.^12^ examined the combined effects of a uniform heat source and nanoparticles on heat transfer in a fluid in a divergent channel placed in a magnetic field. Talib et al.^13^ studied the influence of $\beta - Ga_{2} O_{3}$ nanoparticles on heat transfer in fluid. Sreedevi et al.^14^ studied the simultaneous effects of nanoparticles, thermal radiation, and magnetic field on heat transfer in a fluid-filled square cavity. Nadooshan et al.^15^ discussed the improvement in viscosity of the fluid due to the dispersion of hybrid nanoparticles. They also analyzed the thermal efficiency of the fluid due to the dispersion of hybrid nanoparticles. Nazir et al.^16^ examined the role of hybrid nanoparticles on heat transfer in Carreau-Yasuda fluid numerically using the finite element method. Asjad et al.^17^ theoretically analyzed the influence of hybrid nanoparticles on thermal enhancement in a non-Newtonian fluid in a channel. Cheng et al.^18^ discussed the role of hybrid nanoparticles on the effectiveness of thermal conductivity of fluid exhibiting elastoviscoplastic rheological behavior. Sarwar et al.^19^, Rehman and Salleh^20^, Nayak et al.^21^, and Alanazi et al.^22^ theoretically proved that hybrid nanoparticles are more effective than mono particles in enhancing the thermal performance of the working fluid. Ejaz et al.^23^ discussed the impact of porous medium on heat transfer and fluid flow subjected to a magnetic field. Nazari et al.^24^ discussed the impact of porous medium on heat transfer in a filled in a lid-driven square cavity. Javaherdeh et al.^25^ numerically analyzed the impact of porous medium on heat transfer in a fluid flowing in a channel. Yaseen et al.^26^ theoretically studied the combined influence of porous medium, thermal radiation, heat generation/absorption and hybrid nanoparticles on heat transfer in fluid between two parallel plates. Shende et al.^27^ explored the combined effects of porous medium, nanoparticles, and the non-Newtonian nature of fluid on heat transfer. Ragavi et al.^28^ numerically analyzed the impact of nanoparticles on heat transfer in Casson fluid subjected to a slip mechanism. Ragavi et al.^29^ numerically discussed the impact of nanoparticles on heat transfer in Williamson fluid in a porous medium. Sreenivasulu et al.^30^ also analyzed the impact of nanoparticles on heat transfer in upper convective Maxwell fluid under the influence of an inclined magnetic field.

The latest studies have revealed that the effectiveness of multi-nanoparticles towards optimal heat transfer is higher than that of single nanoparticles. Owing to this positive aspect of the role of hybrid nanoparticles, numerous advancements have been made. The use of hybrid nanoparticles in processes involving optimal heat transfer through enhanced characteristics like stability, durability, and compatibility, etc. This is a core reason that researchers have been focusing on this area of research. The research on the role of hybrid nanoparticles is diverse. However, in fluid flow and heat transfer, some investigations can be mentioned here. The influence of Darcy–Forchheimer porous media on heat transfer in fluids can be seen in references^31–37^. Moreover, Sreenivasulu et al.^38^ analyzed the role of hybrid nanoparticles in enhancing the thermal performance of the fluid used for transporting heat. Vinothkumar et al.^39^ studied the role of hybrid nanoparticles in increasing thermal properties. Ragavi et al.^40^ and Ragavi et al.^41^ also analyzed an optimized heat transfer in fluid due to hybrid nanoparticles.

The Lorentz force (that opposes fluid motion and lowers flow velocity) is produced when a magnetic field is applied to an electrically conducting fluid. This resistance increases fluid friction and accelerates the Joule heating phenomena, which is the process by which electrical energy is transformed into thermal energy. This generated heat becomes part of the temperature of the fluid. Consequently, the fluid temperature increases, resulting in a reduction in heat transfer rate. Thus, in magnetohydrodynamic (MHD) fluid flows, magnetic fields have a major impact on both momentum and thermal transfer. Such fluid flows under the influence of a magnetic field are magnetohydrodynamic flow or MHD flow. Several studies investigating heat transfer in MHD flows have been published. For instance, see references^42–47^.

Novelty of this work stems from the following bases (i) it is very important to note that the term $\:-\frac{{\mu\:}_{app}}{{k}^{*}}\mathbf{V}$^ 48^ is a force by the Darcy porous medium, which involves apparent viscosity ($\:{\mu\:}_{app}$), which is, in most of the studies, not considered in the case of the non-Newtonian fluids. It is also worth mentioning that the terms $\:\frac{{\mu\:}_{app}}{{k}^{*}}\left|\mathbf{V}\right|\mathbf{V}\:$and $\:\rho\:F\left|\mathbf{V}\right|\mathbf{V}\bullet\:\mathbf{V}$^48^ are the dissipation terms caused by the Darcy and the Forchheimer porous media, which is ignored in most of the published work. Moreover, in the terms $\:\frac{{\mu\:}_{app}}{{k}^{*}}\left|\mathbf{V}\right|\mathbf{V}\:$ and $\:\frac{{\mu\:}_{app}}{{k}^{*}}\mathbf{V}$, apparent viscosity is also considered, which is considerable in the case of non-Newtonian fluids. Thus, the term $\:\left({\mu\:}_{tnf}+\frac{{\tau\:}_{y}}{\frac{\partial\:u}{\partial\:y}}\left(1-{e}^{-m\frac{\partial\:u}{\partial\:y}}\right)\right)\left[{\left(\frac{\partial\:u}{\partial\:y}\right)}^{2}+\frac{{u}^{2}}{{k}^{*}}\right]$, is the dissipation due to the viscous and Dracy porous medium force, and this dissipation term can be reduced to $\:{\mu\:}_{tnf}\left[{\left(\frac{\partial\:u}{\partial\:y}\right)}^{2}+\frac{{u}^{2}}{{k}^{*}}\right]$ for the case of Newtonian fluid when $\:{\tau\:}_{y}=0$. Similarly, the Darcy porous medium $\:-\frac{1}{{k}^{*}}\left[{\mu\:}_{tnf}+\frac{{\tau\:}_{y}}{\frac{\partial\:u}{\partial\:y}}\left(1-{e}^{-m\frac{\partial\:u}{\partial\:y}}\right)\right]u$ is calculated from the $\:-\frac{{\mu\:}_{app}}{{k}^{*}}\mathbf{V}$ which can be reduced to $\:-\frac{{\mu\:}_{tnf}}{{k}^{*}}u$ in the case of Newtonian fluid. This is the novelty of this work. This article is divided into five sections. Section 1 is about the literature review and background of this study. Section 2 contains a detailed mathematical formulation. A mathematical formulation is given in Sect. 3. The results based on the numerical simulations are discussed in Sect. 4. The concluding summary is given in Sect. 5.

## Problem statement and formulation

**Fig. 1:**
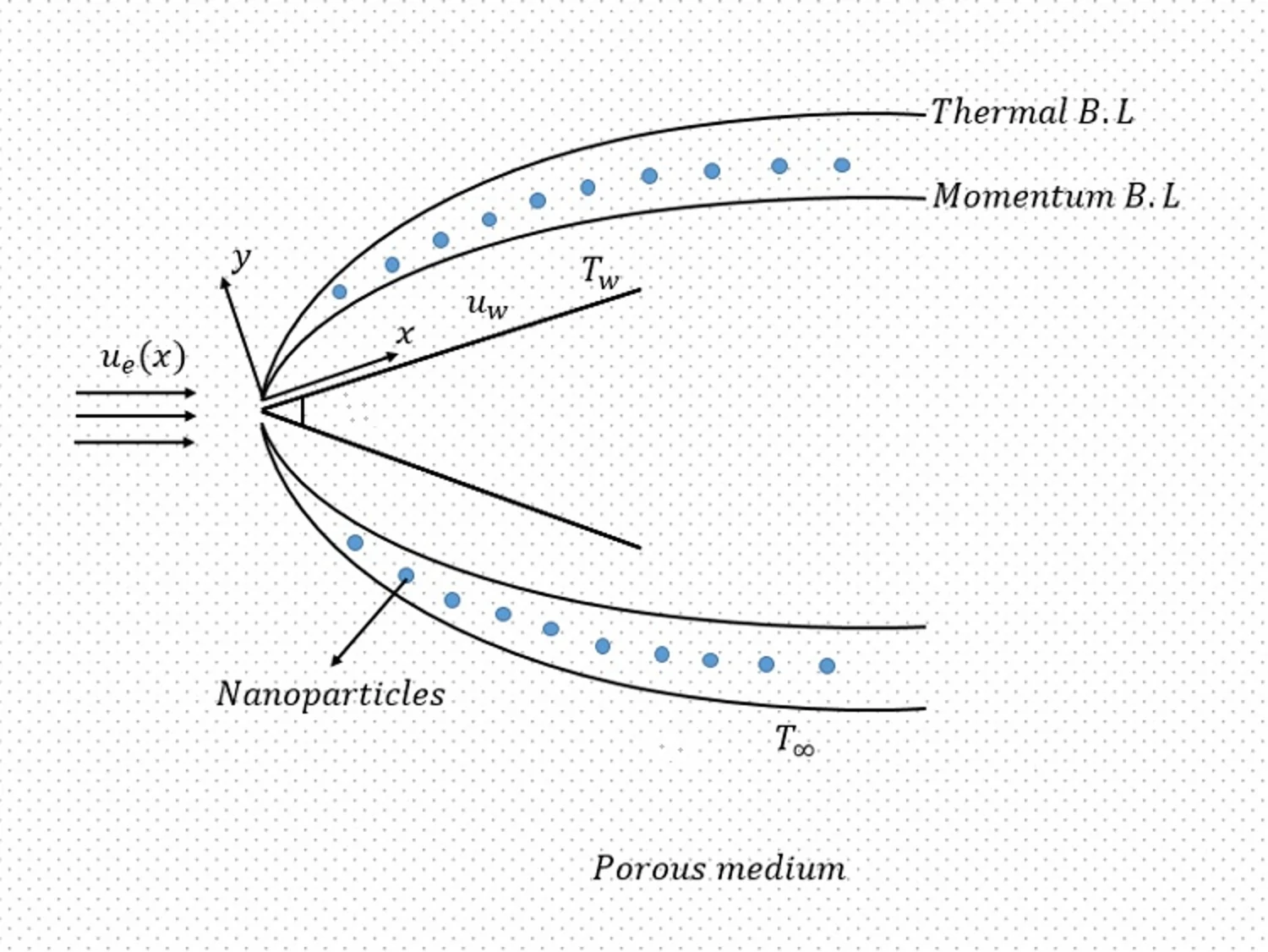
Graphical representation of fluid flow problem and coordinate system.

Let us consider heat transfer in a non-Newtonian fluid (Bingham fluid) over a heated wedge kept in a porous medium subjected to an applied magnetic field. The nanoparticles (of three types) are considered for the improvement of the thermal conductivity of the viscoplastic fluid. Free stream flow has a non-uniform velocity $\:{U}_{e}\left(x\right)=b{x}^{n}.$The walls of the wedge have a variable temperature $\:{T}_{w}\left(x\right)={T}_{\infty\:}+c{x}^{n}$, where $\:{T}_{\infty\:}$ is the ambient temperature, and $\:c\:$ is a constant with units $\:\frac{K}{{L}^{n}}$. The viscous dissipation and thermal radiation are taken into account. The physical model is shown in Fig. 1.

The following assumptions are considered while formulating the mathematical models.

- Fluid is a Bingham fluid, and its rheological model is Bingham-Papanastasiou.
- The free stream velocity is nonlinear and is defined by $\:{U}_{e}\left(x\right)=b{x}^{n},$ and the wall velocity is defined by $\:{U}_{w}\left(x\right)=a{x}^{n}$, where * a* and $\:\:b$ are constant and have units $\:\frac{{m}^{-\left(n+1\right)}}{sec}$.
- Fluid flow is steady and two-dimensional, and incompressible.
- Bingham fluid has the property to emit thermal radiation. Viscous dissipation is significant.
- The temperature of the wall of the wedge varies in a nonlinear manner, expressed by $\:{T}_{w}\left(x\right)=\:{T}_{\infty\:}+c{x}^{n}$.
- Bingham fluid has constant thermal conductivity with respect to temperature and pressure. However, it depends on the thermal conductivity of nanoparticles.
- Fluid flow and flow of heat are steady, and two-dimensional Bingham fluid flow is incompressible.
- Boundary layer approximations are valid.

The governing laws^49^, respectively, are the laws of conservation of mass, momentum, and energy, which are given by.

### Continuity equation

a mathematical form of the law of conservation of mass.

1 $$\:\nabla\:\cdot\:\mathbf{V}=0,$$

### Momentum equation

a mathematical form of the law of conservation of linear momentum.

2 $$\:{\rho\:}_{tnf}\frac{\partial\:\mathbf{V}}{\partial\:t}\:=\:-\:\nabla\:P\:+\:\nabla\:\cdot\:\boldsymbol{\tau\:}-\frac{{\mu\:}_{app}}{{k}^{*}}\mathbf{V}-{\rho\:}_{tnf}F\left|\mathbf{V}\right|\mathbf{V}\:+\:\mathbf{J}\times\:\mathbf{B},$$

### Energy equation

a mathematical form of the law of conservation of heat energy.

3 $$\:{\left(\rho\:{C}_{p}\right)}_{tnf}\frac{\partial\:T}{\partial\:t}\:=\:k{\nabla\:}^{2}T\:+{\upphi\:}+\frac{1}{{\sigma\:}_{tnf}}\mathbf{J}\bullet\:\mathbf{J}+\:\nabla\:\bullet\:{q}_{r}\:+\frac{{\mu\:}_{app}}{{k}^{*}}\left|\mathbf{V}\right|\mathbf{V}+{{\rho\:}_{tnf}}F\left|\mathbf{V}\right|\mathbf{V}\bullet\:\mathbf{V}.$$

where $\:{\upphi\:}\:$is the viscous dissipation term, $\:{\mu\:}_{app}$ is the apparent viscosity of Bingham-Papanastasiou, $\:\boldsymbol{V}$ is the velocity vector, $\:\boldsymbol{B}$ is the magnetic field, $\:\boldsymbol{J}$ is the current density, $\:{\boldsymbol{q}}_{\boldsymbol{r}}$ is a thermal radiation heat flux vector, which is defined by $\:{\boldsymbol{q}}_{\boldsymbol{r}}=-\frac{16{\sigma\:} ^{*}{T}_{\infty\:}^{3}}{3{k}_{1}}\:\nabla\:T.$

The viscoplastic fluid characterized by the rheological model^50–56^, called the Bingham–Papanastasiou model, is given by

4 $$\:\boldsymbol{\tau\:}={\mu\:}_{app}{A}_{1}=\left[{\mu\:}_{p}+\frac{{\tau\:}_{y}}{\dot{\gamma\:}}\left(1-{e}^{-m\dot{\gamma\:}}\right)\right]{A}_{1}$$

where $\:{\tau\:}_{y}$ is yield stress,$\:\:\dot{\gamma\:}$ is Shear rate, $\:{A}_{1}$ is the Revilon-Erickson tensor,* m* is the growth parameter,$\:\:{\mu\:}_{p}$ is the plastic viscosity.

### Boundary layer approximation

The fundamental aspects of momentum, heat, in the Bingham viscoplastic nanofluid within a porous medium are sufficiently captured by the coupled system of nonlinear boundary layer equations that results from the reduction of the conservation of momentum and energy equations under these assumptions, according to the following order of magnitudes.

$$\:O\left(\sigma\:\right)=O\left(T\right)=O\left(C\right)=O\left({C}_{p}\right)=O\left({k}_{r}\right)=O\left(\rho\:\right)=O\left(F\right)=1$$, $$\:O\left(u\right)=\delta\:$$, $$\:O\left(r\right)=O\left(m\right)=\delta,$$ $$\:O\left(\mu\:\right)=O\left({k}^{*}\right)=O\left(K\right)=O\left(D\right)={\delta\:}^{2}$$, $$\:O\left({\tau\:}_{y}\right)=\delta\:$$, $$\:O\left(w\right)=O\left(z\right)=1$$.

Hence, the boundary-layer approximations simplify the Eqs. (1)-(4), and these simplified equations are given by.

*Continuity equation*

5 $$\:\frac{\partial\:u}{\partial\:x}+\frac{\partial\:v}{\partial\:y}=0,$$

*Momentum equation*

6 $$\begin{aligned} \rho _{{tnf}} \left( {u\frac{{\partial u}}{{\partial x}} + v\frac{{\partial u}}{{\partial y}}} \right) & = \rho _{{tnf}} \left( {U_{e} \frac{{\partial U_{e} }}{{\partial x}}} \right) + \sigma _{{tnf}} B_{0}^{2} \left( {U_{e} - u} \right) + \left[ {\frac{{\left( {\mu _{{tnf}} + m\tau _{y} } \right)}}{{k^{*} }}} \right]U_{e} - \rho _{{tnf}} F\left( {u^{2} - U_{e}^{2} } \right) \\ & + \left( {\mu _{{tnf}} + m\tau _{y} e^{{ - m\frac{{\partial u}}{{\partial y}}}} } \right)\frac{{\partial ^{2} u}}{{\partial y^{2} }} - \frac{1}{{k^{*} }}\left[ {\mu _{{tnf}} + \frac{{\tau _{y} }}{{\frac{{\partial u}}{{\partial y}}}}\left( {1 - e^{{ - m\frac{{\partial u}}{{\partial y}}}} } \right)} \right]u, \\ \end{aligned}$$

*Energy equation*

7 $$\begin{gathered} \left( {\rho C_{p} } \right)_{{tnf}} \left[ {u\frac{{\partial T}}{{\partial x}} + v\frac{{\partial T}}{{\partial y}}} \right] = k_{{tnf}} \frac{{\partial ^{2} T}}{{\partial y^{2} }} + \rho _{{tnf}} Fu^{2} + \sigma _{{tnf}} B_{0}^{2} u \hfill \\ + \left( {\mu _{{tnf}} + \frac{{\tau _{y} }}{{\frac{{\partial u}}{{\partial y}}}}\left( {1 - e^{{ - m\frac{{\partial u}}{{\partial y}}}} } \right)} \right)\left[ {\left( {\frac{{\partial u}}{{\partial y}}} \right)^{2} + \frac{{u^{2} }}{{k^{*} }}} \right] + \frac{{16\sigma ^{*} ~T_{\infty }^{3} }}{{3k_{1} }}\frac{{\partial ^{2} T}}{{\partial y^{2} }}, \hfill \\ \end{gathered}$$

It is very important to note that the term $\:-\frac{{\mu\:}_{app}}{{k}^{*}}\mathbf{V}$^48^ is a force by the Darcy porous medium, which involves apparent viscosity ($\:{\mu\:}_{app}$), which is, in most of the studies, not considered in the case of the non-Newtonian fluids. It is also worth mentioning that the 4th and 5th terms on the right-hand side of Eq. (3) $\:\frac{{\mu\:}_{app}}{{k}^{*}}\left|\mathbf{V}\right|\mathbf{V}\:$and $\:\rho\:F\left|\mathbf{V}\right|\mathbf{V}\bullet\:\mathbf{V}$^48^ the dissipation caused by Darcy and Forchheimer porous medium, which is ignored in most of the published work. Moreover, in the terms $\:\frac{{\mu\:}_{app}}{{k}^{*}}\left|\mathbf{V}\right|\mathbf{V}\:$ and $\:\frac{{\mu\:}_{app}}{{k}^{*}}\mathbf{V}$, apparent viscosity is also considered, which is considerable in the case of non-Newtonian fluids. Thus, the term $\:\left({\mu\:}_{tnf}+\frac{{\tau\:}_{y}}{\frac{\partial\:u}{\partial\:y}}\left(1-{e}^{-m\frac{\partial\:u}{\partial\:y}}\right)\right)\left[{\left(\frac{\partial\:u}{\partial\:y}\right)}^{2}+\frac{{u}^{2}}{{k}^{*}}\right]$, is the dissipation due to the viscous and Dracy porous medium force, and this dissipation term can be reduced to $\:{\mu\:}_{tnf}\left[{\left(\frac{\partial\:u}{\partial\:y}\right)}^{2}+\frac{{u}^{2}}{{k}^{*}}\right]$ for the case of Newtonian fluid when $\:{\tau\:}_{y}=0$. Similarly, the Darcy porous medium $\:-\frac{1}{{k}^{*}}\left[{\mu\:}_{tnf}+\frac{{\tau\:}_{y}}{\frac{\partial\:u}{\partial\:y}}\left(1-{e}^{-m\frac{\partial\:u}{\partial\:y}}\right)\right]u$ is calculated from the $\:-\frac{{\mu\:}_{app}}{{k}^{*}}\mathbf{V}$ which can be reduced to $\:-\frac{{\mu\:}_{tnf}}{{k}^{*}}u$ in the case of Newtonian fluid. This is the novelty of this work, as mentioned in the last paragraph of the introduction.

In fluid mechanics, the no-slip theory is fundamental for developing boundary conditions. This theory states that the fluid velocity at a solid surface is equal to the surface velocity. And the temperature of the fluid at the solid surface is equal to the temperature of the surface. It follows that this shows that the tangential and normal components of the fluid velocity are equivalent to those of the solid’s velocity. According to this hypothesis, the fluid’s temperature at the solid surface is the same as the wall’s temperature. The boundary conditions are

8 $$\:\left.\begin{array}{c}u\left(x,0\right)=\:{U}_{w}\left(x\right)=a{x}^{n},\:\:\:v\left(x,0\right)=0,\:\:T\left(x,0\right)={T}_{w}={T}_{\infty\:}+c{x}^{n}\\\:u\left(x,\infty\:\right)\to\:{U}_{e}\left(x\right),\:\:v\left(x,\infty\:\right)\to\:0,\:\:\:\:T\left(x,\infty\:\right)\to\:{T}_{\infty\:}\end{array}\right\}.$$

To make Eqs. (5) -(8), following similarity transformation is used

9 $$\:\left.\begin{array}{c}\eta\:=\sqrt{\frac{{U}_{w}\left(x\right)}{x{\nu\:}_{f}}\:}y,\:\:\:T={T}_{\infty\:}+\left({T}_{w}-{T}_{\infty\:}\right)\theta\:\left(\eta\:\right)\\\:u={U}_{w}{f}^{{\prime\:}}\left(\eta\:\right),\:\:\:v=-\frac{1}{2}\:\sqrt{a{\nu\:}_{f}{x}^{n-1}}\:\left[\left(n+1\right)f\left(\eta\:\right)+\left(n-1\right)\eta\:{f}^{{\prime\:}}\left(\eta\:\right)\right]\end{array}\right\}.$$

Hence, one gets

10 $$\begin{aligned} A_{1} nA^{2} & + \left[ {A_{2} + Bnm^{*} \exp \left( { - m^{*} \sqrt {\mathrm{Re} } f^{\prime\prime}~} \right)} \right]f^{\prime\prime\prime}~~ - A_{3} M\left( {f^{\prime} - A} \right) - A_{1} Fr\left( {f^{{'2}} - A^{2} } \right) \\ & - Da\left[ {A_{2} + \frac{{Bn}}{{\sqrt {\mathrm{Re} } ~f''}}~\left( {1 - \exp \left( { - m^{*} \sqrt {\mathrm{Re} } f^{\prime\prime}} \right)} \right)} \right]f^{\prime} \\ & + ADa\left( {A_{2} + m^{*} Bn} \right) - A_{1} \left( {nf^{{'2}} - \frac{{n + 1}}{2}ff^{\prime\prime}} \right) = 0, \\ \end{aligned}$$

11 $$\begin{aligned} \theta ^{\prime\prime}\frac{{A_{5} }}{{\Pr }} & + A_{3} MEc\left( {f^{\prime}} \right)^{2} + A_{1} FrEcf^{{'3}} + \frac{4}{3}R\theta ^{\prime\prime} - A_{4} \left( {\theta nf^{\prime} - \frac{{n + 1}}{2}\theta ^{\prime}f} \right) \\ & + \left( {Ecf^{{''2}} + EcDaf^{{'2}} } \right)\left[ {A_{2} + \frac{{Bn}}{{\sqrt {\mathrm{Re} } f^{\prime\prime}}}\left( {1 - \exp \left( { - m^{*} \sqrt {\mathrm{Re} } f^{\prime\prime}} \right)} \right)} \right] = 0. \\ \end{aligned}$$

and a set of boundary conditions are

12 $$\:f\left(0\right)=0,{f}^{{\prime\:}}\left(0\right)=1,\theta\:\left(0\right)=1,{f}^{{\prime\:}}\left(\infty\:\right)\to\:A,\theta\:\left(\infty\:\right)\to\:0,$$

In the above ODEs, the parameters $\:{A,\:A}_{1},\:{A}_{2},\:{A}_{3},{A}_{4},{A}_{5}$ $\:Re,\:Bn,\:Pr,\:Ec,\:M,\:Da,\:Fr$, * R* and $\:{m}^{*}$are expressed by:

$$\begin{gathered} A = \frac{b}{a},\,A_{1} = \frac{{\rho _{{tnf}} }}{{\rho _{f} }},~A_{2} = \frac{{\mu _{{tnf}} }}{{\mu _{f} }},~A_{3} = \frac{{\sigma _{{tnf}} }}{\sigma _{{f}} },~A_{4} = \frac{{\left( {\rho C_{p} } \right)_{{tnf}} }}{{\left( {\rho c_{p} } \right)_{f} }},~A_{5} = \frac{{k_{{tnf}} }}{{k_{f} }},\mathrm{Re} = \frac{{ax^{{n + 1}} }}{{\nu _{f} }},Bn = \frac{{\tau _{y} }}{{\mu _{f} \left( {ax^{{n - 1}} } \right)}}, \hfill \\ \Pr = \frac{{\left( {\mu C_{p} } \right)_{{f}} }}{k_{{f}}},Ec = \frac{{a^{2} x^{n} }}{{c\left( {C_{p} } \right)_{{f}} }},Fr = \frac{{C_{b} }}{{\sqrt {k^{*} } }},Da = \frac{\left( {\nu} \right)_{{f}} }{{k^{*} ax^{{n - 1}} }},M = \frac{{B_{o}^{2} }}{{\rho _{f} \left( {ax^{{n - 1}} } \right)}},R = \frac{{4\sigma ^{*} T_{\infty }^{3} }}{{k_{1} k}},~m^{*} = max ^{{n - 1}} \hfill \\ \end{gathered}$$

These are respectively called the stretching parameter (* A*), Thermo-physical constant ($\:{A}_{1}\:to\:{A}_{5}$), Reynolds number (* Re*), Bingham number (* Bn*), Prandtl number (* Pr*), Eckert number (* Ec*), Forchheimer (* Fr*), Darcy parameter (* Da*), Hartmann number (* M*), Radiation parameter (* R*) and Growth parameter ($\:{m}^{*}$).

The quantities which provide insight into the heat transport phenomena and stress analysis are Nusselt number (* Nu*) and skin friction ($\:{C}_{f}$) coefficient, which are mathematically defined below:

13 $$\:{C}_{f}\:=\frac{{\tau\:}_{xy}}{{\rho\:}_{f}{u}_{w}^{2}},\:Nu\:=\frac{x{q}_{w}}{{k}_{f}\left({T}_{w}-{T}_{\infty\:}\right)}$$

where

$$\:{\tau\:}_{xy}={\left[\frac{\partial\:u}{\partial\:y}{\mu\:}_{tnf}+{\tau\:}_{y}\left(1-{e}^{-m\frac{\partial\:u}{\partial\:y}}\right)\right]}_{y=0}\:,\:{q}_{w}={\left[-\left({k}_{tnf}\right)\frac{\partial\:T}{\partial\:y}+{q}_{r}\right]}_{y=0}$$

Hence one gets the normalized forms of skin friction and the Nusselt number which are

14 $$\:{C}_{f}\:=\frac{1}{\sqrt{Re}}\:\left[{A}_{2}f^{\prime\prime}\left(0\right)+\frac{Bn}{\sqrt{Re}}\:\left(1\:-\:{e}^{-{m}^{*}\sqrt{Re}f^{\prime\prime}\left(0\right)\:}\right)\right],$$

15 $$\:Nu\:=-\sqrt{Re}\:\left({A}_{5}+\frac{4}{3}R\right){\theta\:}^{{\prime\:}}\left(0\right)$$

The numerical values of properties of Ternary nanofluid and nanoparticles are given in Table 1, whereas the relations between properties of base fluid and nanoparticles are given in Table 2.

**Table 1:** Thermophysical values of base fluid and nano-particles^57,58^.

Physical properties	* SA*	$\:\mathrm{C}\mathrm{u}$	$\:F{e}_{3}{O}_{4}$	$\:\mathrm{G}\mathrm{O}$
$\:C_{p}(J/Kgk)$	* 3630*	* 385*	* 397.21*	* 717*
$\:\rho\:(Kg/{m}^{3})$	* 1063.8*	* 8933*	* 5060*	* 1800*
$\:K\:(W/m\:k)$	* 0.387*	* 401*	* 904.4*	* 5000*
$\:\sigma\:{\left({\Omega\:}m\right)}^{-1}$	$\:9.75\times\:{10}^{-5}$	$\:5.96\times\:{10}^{7}$	$\:2.09\times\:{10}^{4}$	$\:6.30\times\:{10}^{7}$

**Table 2:** Relations between thermo-physical properties of base fluid and nano-particles^59^.

Properties	Ternary nanofluid
Density	$\:{\rho\:}_{tnf}=(1-{\phi\:}_{1})\left\{\right(1-{\phi\:}_{2}\left)\right[(1-{\phi\:}_{3}){\rho\:}_{F}+{\phi\:}_{3}{\rho\:}_{3}]+{\phi\:}_{2}{\rho\:}_{2}\}+{\phi\:}_{1}{\rho\:}_{1}$.
Heat capacity	$\:{\left({C}_{p}\right)}_{tnf}=(1-{\phi\:}_{1})\left\{\right(1-{\phi\:}_{2}\left)\right[(1-{\phi\:}_{3}){\left({C}_{p}\right)}_{F}+{\phi\:}_{3}{\left({C}_{p}\right)}_{3}]+{\phi\:}_{2}{\left({C}_{p}\right)}_{2}\}+{\phi\:}_{1}{\left({C}_{p}\right)}_{1}$.
Viscosity	$\:{\mu\:}_{tnf}=\frac{\mu\:f}{(1-{\phi\:}_{1}{)}^{2.5}(1-{\phi\:}_{2}{)}^{2.5}(1-{\phi\:}_{3}{)}^{2.5}}$.
Thermal conductivity	$\:{K}_{tnf}=\left[\frac{{K}_{1}+2{K}_{hnf}-2{\phi\:}_{1}\left({K}_{hnf}-{K}_{1}\right)}{{K}_{1}+2{K}_{hnf}+{\phi\:}_{1}\left({K}_{hnf}-{K}_{1}\right)}\right]\left[\frac{{K}_{2}+2{K}_{nf}-2{\phi\:}_{2}({K}_{nf}-{K}_{2})}{{K}_{2}+2{K}_{nf}+{\phi\:}_{2}({K}_{nf}-{K}_{2})}\right]\left[\frac{{K}_{3}+2{K}_{f}-2{\phi\:}_{3}({K}_{f}-{K}_{3})}{{K}_{3}+2{K}_{f}+{\phi\:}_{3}({K}_{f}-{K}_{3})}\right]$.
Electrical conductivity	$\:{\sigma\:}_{tnf}=\left[\frac{{\sigma\:}_{1}+2{\sigma\:}_{hnf}-2{\phi\:}_{1}({\sigma\:}_{hnf}-{\sigma\:}_{1})}{{\sigma\:}_{1}+2{\sigma\:}_{hnf}+{\phi\:}_{1}({\sigma\:}_{hnf}-{\sigma\:}_{1})}\right]\left[\frac{{\sigma\:}_{2}+2{\sigma\:}_{nf}-2{\phi\:}_{2}({\sigma\:}_{nf}-{\sigma\:}_{2})}{{\sigma\:}_{2}+2{\sigma\:}_{nf}+{\phi\:}_{2}({\sigma\:}_{nf}-{\sigma\:}_{2})}\right]\left[\frac{{\sigma\:}_{3}+2{\sigma\:}_{f}-2{\phi\:}_{3}({\sigma\:}_{f}-{\sigma\:}_{3})}{\sigma\:3+2{\sigma\:}_{f}+{\phi\:}_{3}({\sigma\:}_{f}-{\sigma\:}_{3})}\right]$.

## Numerical method

Several numerical methods can be used for solving boundary value problems related to computational fluid dynamics (CFD). However, while applying a numerical method, the accuracy and convergence are at the first preference. In this regard, GFEM is the most qualifying method. Therefore, this method is used for the numerical solution of developed boundary value problems. The steps involved in the implementation of GFEM are described below:

I. Weighted residuals are multiplied by weight functions and integrated over a particular line element. The weights are taken equal to linear shape functions (write here shape functions). Thus, the weighted residua
  16 $$\:{\int\:}_{{\eta\:}_{e}}^{{\eta\:}_{e+1}}w\left(f^{\prime\:}-h\right)d\eta\:=0,$$
  17 $$\mathop \smallint \limits_{{\eta _{e} }}^{{\eta _{{e + 1}} }} w\left[ \begin{gathered} h^{\prime\prime}\left( {A_{2} + m^{*} Bn~e^{{ - m^{*} \sqrt {\mathrm{Re} } h^{\prime}}} } \right) - nA_{1} \left( {h^{2} - A^{2} } \right) - A_{3} M\left( {h - A} \right) + \frac{{n + 1}}{2}fh^{\prime} \hfill \\ - A_{1} Fr\left( {h^{2} - A^{2} } \right) - Da\left[ {A_{2} + \frac{{Bn}}{{\sqrt {\mathrm{Re} } ~h^{\prime}}}~\left( {1 - e^{{ - m^{*} \sqrt {\mathrm{Re} } h^{\prime}}} } \right)} \right]h + ADa\left( {A_{2} + m^{*} Bn} \right) \hfill \\ \end{gathered} \right]d\eta = 0,$$
  18 $$\mathop \smallint \limits_{{\eta _{e} }}^{{\eta _{{e + 1}} }} w\left[ \begin{gathered} \left[ {\left( {\frac{{A_{5} }}{{\Pr }} + \frac{4}{3}R} \right)\theta ^{\prime\prime} + A_{3} MEch^{2} } \right. + A_{1} FrEch^{3} - A_{4} \left( {nh\theta - \frac{{n + 1}}{2}f\theta ^{\prime}} \right) \hfill \\ + \left( {Ech^{{'2}} + EcDah^{2} } \right)\left[ {A_{2} + \frac{{Bn}}{{\sqrt {\mathrm{Re} } h^{\prime}}}\left( {1 - e^{{ - m^{*} \sqrt {\mathrm{Re} } h'}} } \right)} \right] \hfill \\ \end{gathered} \right]d\eta = 0.$$
II. The weak forms of the above residuals can be obtained by integrating the highest order linear terms.
  19 $$\:{\int\:}_{{\eta\:}_{e}}^{{\eta\:}_{e+1}}w\left(f^{\prime\:}-h\right)d\eta\:=0,$$
  20 $$\begin{aligned} \mathop \smallint \limits_{{\eta _{e} }}^{{\eta _{{e + 1}} }} & \left[ \begin{gathered} - \left( {A_{2} + m^{*} Bn~e^{{ - m^{*} \sqrt {\mathrm{Re} } h^{\prime}}} } \right)w'h^{\prime} - nA_{1} \left( {h^{2} - A^{2} } \right)w - A_{3} M\left( {h - A} \right)w + \frac{{n + 1}}{2}wfh^{\prime} \hfill \\ - A_{1} Fr\left( {h^{2} - A^{2} } \right)w - Da\left[ {A_{2} + \frac{{Bn}}{{\sqrt {\mathrm{Re} } ~h^{\prime}}}~\left( {1 - e^{{ - m^{*} \sqrt {\mathrm{Re} } h^{\prime}}} } \right)} \right]wh + ADa\left( {A_{2} + m^{*} Bn} \right)w \hfill \\ \end{gathered} \right]d\eta \\ & = \int\limits_{\Gamma } { - \left( {A_{2} + m^{*} Bn~e^{{ - m^{*} \sqrt {\mathrm{Re} } h^{\prime}}} } \right)wh^{\prime}d\Gamma}, \\ \end{aligned}$$
  21 $$\begin{aligned} \mathop \smallint \limits_{{\eta _{e} }}^{{\eta _{{e + 1}} }} & \left[ \begin{gathered} - \left( {\frac{{A_{5} }}{{\Pr }} + \frac{4}{3}R} \right)w'\theta ^{\prime} + A_{3} MEcwh^{2} + A_{1} FrEcwh^{3} - A_{4} w\left( {nh\theta - \frac{{n + 1}}{2}f\theta ^{\prime}} \right) \hfill \\ + w\left( {Ech^{{'2}} + EcDah^{2} } \right)\left[ {A_{2} + \frac{{Bn}}{{\sqrt {\mathrm{Re} } h^{\prime}}}\left( {1 - e^{{ - m^{*} \sqrt {\mathrm{Re} } h'}} } \right)} \right] \hfill \\ \end{gathered} \right]d\eta \\ & = \int\limits_{\Gamma } { - \left( {\frac{{A_{5} }}{{\Pr }} + \frac{4}{3}R} \right)w\theta ^{\prime}d\Gamma }. \\ \end{aligned}$$
III. The Galerkin approximations are substituted in the weak forms, and stiffness elements are calculated.
  22 $$\:{K}_{ij}^{11}={\int\:}_{{\eta\:}_{e}}^{{\eta\:}_{e+1}}\left({\psi\:}_{i}\frac{d{\psi\:}_{j}}{d\eta\:}\right)d\eta\:,\:{K}_{ij}^{12}=-{\int\:}_{{\eta\:}_{e}}^{{\eta\:}_{e+1}}\left({\psi\:}_{i}{\psi\:}_{j}\right)d\eta\:,\:{K}_{ij}^{13}=0,$$
  23 $$K_{{ij}}^{{22}} = \mathop \smallint \limits_{{\eta _{e} }}^{{\eta _{{e + 1}} }} \left[ \begin{gathered} - \left( {A_{2} + m^{*} Bn~e^{{ - m^{*} \sqrt {\mathrm{Re} } \bar{h}^{'} }} } \right)\frac{{d\psi _{i} }}{{d\eta }}\frac{{d\psi _{j} }}{{d\eta }} - nA_{1} \left( {\bar{h}\psi _{j} - A^{2} } \right)\psi _{i} - A_{3} M\left( {\psi _{j} - A} \right)\psi _{i} + \frac{{n + 1}}{2}\bar{f}\psi _{i} \frac{{d\psi _{j} }}{{d\eta }} \hfill \\ - A_{1} Fr\left( {\bar{h}\psi _{j} - A^{2} } \right)\psi _{i} + ADa\left( {A_{2} + m^{*} Bn} \right)\psi _{i} - Da\left( {A_{2} + \frac{{Bn}}{{\sqrt {\mathrm{Re} } \bar{h}^{'} }}~\left( {1 - e^{{ - m^{*} \sqrt {\mathrm{Re} } \bar{h}^{'} }} } \right)} \right)\psi _{i} \psi _{j} \hfill \\ \end{gathered} \right]d\eta,$$
  24 $$\:{K}_{ij}^{21}={K}_{ij}^{23}={K}_{ij}^{31}=0,$$
  25 $$K_{{ij}}^{{32}} = \mathop \smallint \limits_{{\eta _{e} }}^{{\eta _{{e + 1}} }} \left[ \begin{gathered} A_{3} MEc\bar{h}\psi _{i} \psi _{j} + A_{1} FrEc\bar{h}^{2} \psi _{i} \psi _{j} \hfill \\ + Ec\left[ {A_{2} + \frac{{Bn}}{{\sqrt {\mathrm{Re} } \bar{h}'}}\left( {1 - e^{{ - m^{*} \sqrt {\mathrm{Re} } \bar{h}^{'} }} } \right)} \right]\left( {\bar{h}^{'} \psi _{i} \frac{{d\psi _{j} }}{{d\eta }} + Dah^{2} \bar{h}\psi _{i} \psi _{j} } \right) \hfill \\ \end{gathered} \right]d\eta,$$
  26 $$K_{{ij}}^{{33}} = \int_{{\eta _{e} }}^{{\eta _{{e + 1}} }} {\left[ { - \left( {\frac{{A_{5} }}{{\Pr }} + \frac{4}{3}R} \right)\frac{{d\psi \:_{i} }}{{d\eta \:}}\frac{{d\psi \:_{j} }}{{d\eta \:}} - A_{4} \left( {n\bar{h}\psi \:_{i} \psi \:_{j} - \frac{{n + 1}}{2}\bar{f}\psi \:_{i} \frac{{d\psi \:_{j} }}{{d\eta \:}}} \right)} \right]} d\eta,$$
  27 $$b_{{ij}}^{1} = 0,~b_{{ij}}^{2} = \int\limits_{\Gamma } { - \left( {A_{2} + m^{*} Bn~e^{{ - m^{*} \sqrt {\mathrm{Re} } \bar{h}^{'} }} } \right)\psi _{i} \frac{{d\psi _{j} }}{{d\eta }}d\Gamma },$$
  28 $$b_{{ij}}^{3} = \int\limits_{\Gamma } { - \left( {\frac{{A_{5} }}{{\Pr }} + \frac{4}{3}R} \right)\psi _{i} \frac{{d\psi _{j} }}{{d\eta }}} d\Gamma.$$
IV. The stiffness elements are used to follow the protocol of the assembly process, which results in a nonlinear algebraic system:
  $$\:\boldsymbol{A}\:\boldsymbol{X}=\boldsymbol{B}$$

Where * A* is a coefficient matrix which is a function of unknown $\:\boldsymbol{X}\:~i.e.,\:\boldsymbol{A}=\boldsymbol{A}\left(\boldsymbol{X}\right).$ This requires linearization. The Picard linearization technique is used, and the linear system of algebraic equations is solved numerically with an iterative process.

V. The convergence is ensured through the formula
  $$\left| {X^{{n + 1}} - X^{n} } \right| \le 10^{{ - 5}}$$

where $\:{10}^{-5}$ is the computational tolerance.

VI. Computed solutions are checked to be step-size independent. For this, solutions are computed for different numbers of elements. This analysis is furnished in Table 3, given below.

**Table 3:** Grid-independent analysis for Nusselt number and skin friction when $\:A\:=\:0.2$, $\:n\:=\:0.1$, $\:m\:^{*}=\:0.1$, $\:Bn\:=\:0.2$, $\:Re\:=\:0.5$, $\:M\:=\:0.5$, $\:Da\:=\:0.1$, $\:Fr\:=\:0.2$, $\:R\:=\:0.1$, $\:Pr\:=\:6.45$, $\:Ec\:=\:0.01$, $\:Sc\:=0$, $\:{\phi\:}_{1}=0.15$, $\:{\phi\:}_{2}\:=\:0.05$, $\:{\phi\:}_{3}=0.03$.

No of elements	The Nusselt number	Skin friction coefficient
10	0.6656880802	1.6568314651
30	0.6656880802	1.6568314651
50	0.6656880802	1.6568314651
70	0.6656880802	1.6568314650
90	0.6656880802	1.6568314651
110	0.6656880802	1.6568314651
130	0.6656880802	1.6568314651
150	0.6656880802	1.6568314651
170	0.6656880801	1.6568314651
190	0.6656880801	1.6568314649

VII. The results are validated by comparison with already published data. It is important to mention that the present study reduces to already published benchmarks, Lou et al.^60^, Rehman et al.^61^, and Sharif et al^62^. when $\:A\:=Bn\:=$ $\:Re\:=\:$ $\:M\:=Da=$ $\:Fr\:=$ $\:R\:=$ $\:Ec\:=\:{\phi\:}_{1}=\:{\phi\:}_{2}\:={\phi\:}_{3}=0\:$and $\:n\:=\:1$. This comparison is given in Table 4.

**Table 4:** The verification of results related $$\:\left({Re}^{-\frac{1}{2}}\:Nu\right)$$ by the comparison with published data when $$\:A\:=Bn\:=$$$$\:Re\:=\:$$$$\:M\:=Da=$$$$\:Fr\:=$$$$\:R\:=$$$$\:Ec\:=\:{\phi\:}_{1}=\:{\phi\:}_{2}\:={\phi\:}_{3}=0\:$$and $$\:n\:=\:1$$.

$\:\mathrm{P}\mathrm{r}$	Lou et al.^60^	Rehman et al.^61^	Sharif et al.^62^	Present case
0.7	–	0.80875	–	0.8088342039
1.0	1.0000	1.0000	1.0000	1.0000083736
3.0	1.9238	1.92375	1.9236	1.9236786507
10.0	3.7210	3.72061	3.7206	3.7206711594
100.0	12.2940	12.2940	–	12.2940808212

## Result and discussion

The numerical solutions are computed, and these are further used for the prediction of the behavior of related parameters on flow fields (velocity and temperature) and quantities of engineering interest. The quantities of engineering interest are the wall shear stresses and the Nusselt number. Numerical experiments are performed, and outcomes are visualized and discussed. The quantitative and qualitative predictions are made for quick prediction Bar graphs are displayed and conclusions are made.

### Predictions on the behavior of the wall heat transfer rate

**Fig. 2:**
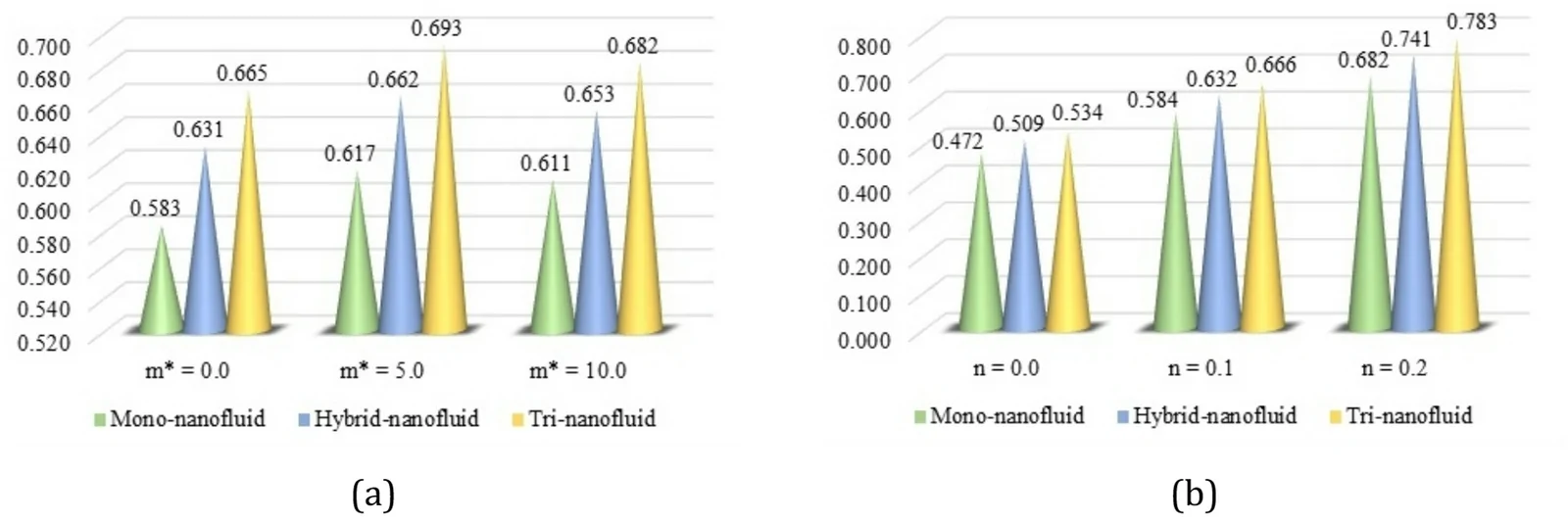
(**a**, **b**) Variation of $\:R{e}_{x}^{-\frac{1}{2}}\:Nu\:$ for mono, hybrid, and tri-nanofluids with different growth parameter $\:\left({m}^{*}\right)$ and power-law index $\:\left(n\right)$, at fixed values of $\:A=0.2,\:Bn=0.2,\:R=0.1,Da=0.1,Fr=0.2,Pr=6.45$, $\:Ec=0.05\:$ and $\:\:M=0.5\:$ considering thermal radiation and heat generation effects.

**Fig. 3:**
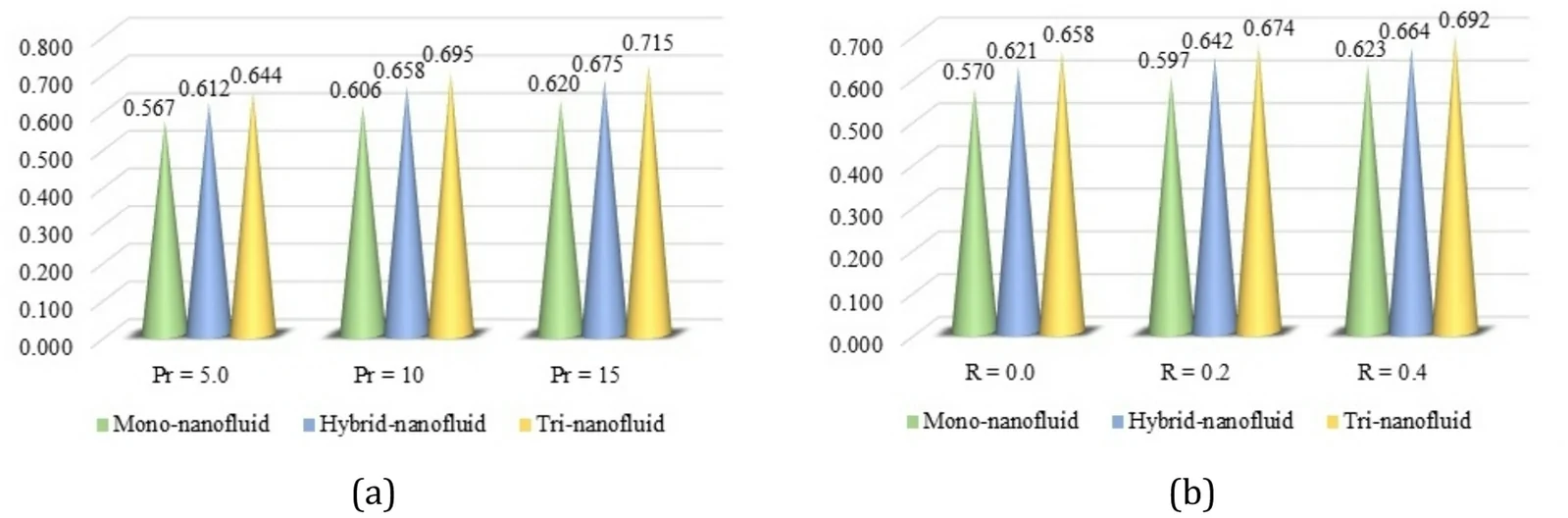
(**a**, **b**) Variation of $\:R{e}_{x}^{-\frac{1}{2}}\:Nu\:$ for mono, hybrid, and tri-nanofluids with different Prandtl number $\left(Pr\right)$ and radiation parameter $\:\left(R\right)$, at fixed values of $\:A=0.2,\:Bn=0.2,\:Da=0.1,Fr=0.2,{m}^{*}=0.1,Ec=0.05\:$ and $\:\:M=0.5\:$ considering thermal radiation and heat generation effects.

**Fig. 4:**
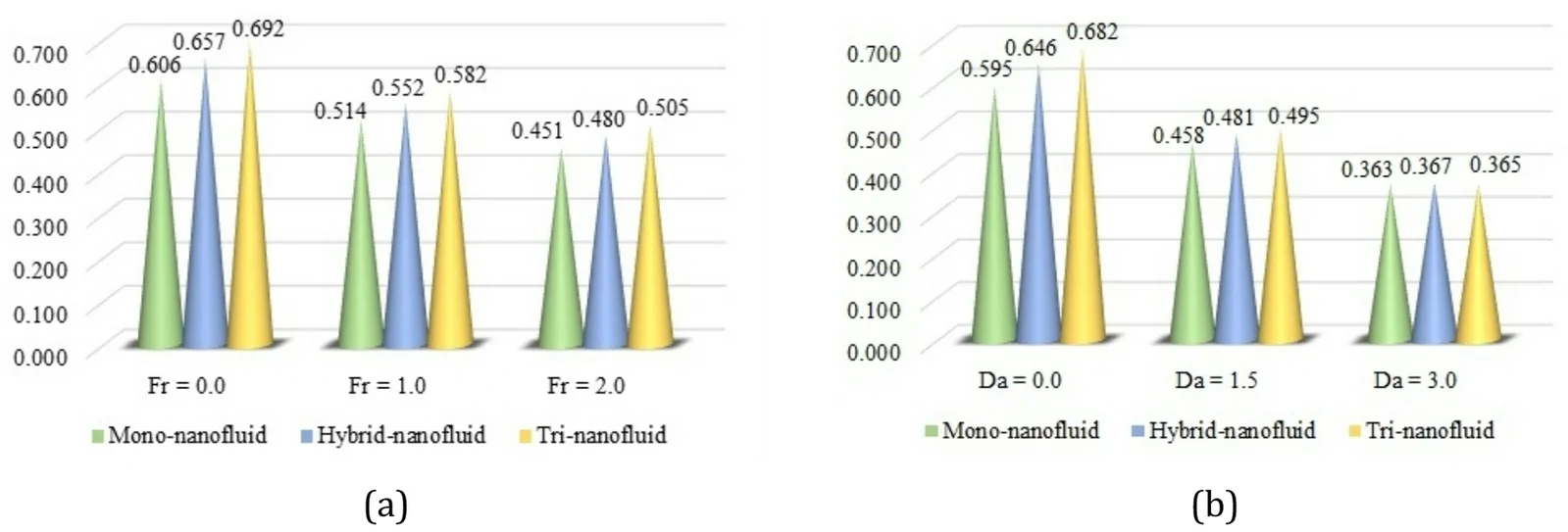
(**a**, **b**) Variation of $\:R{e}_{x}^{-\frac{1}{2}}\:Nu\:$ for mono, hybrid, and tri-nanofluids with different Forchheimer porous medium $\:\left(Fr\right)$ and Darcy number $\:\left(D\right)$, at fixed values of $\:A=0.2,\:Bn=0.2,\:Da=0.1,R=0.1,{m}^{*}=0.1$,$\:Pr=6.45\:~\mathrm{and}~\:Ec=0.05\:$ considering thermal radiation and heat generation effects.

**Fig. 5:**
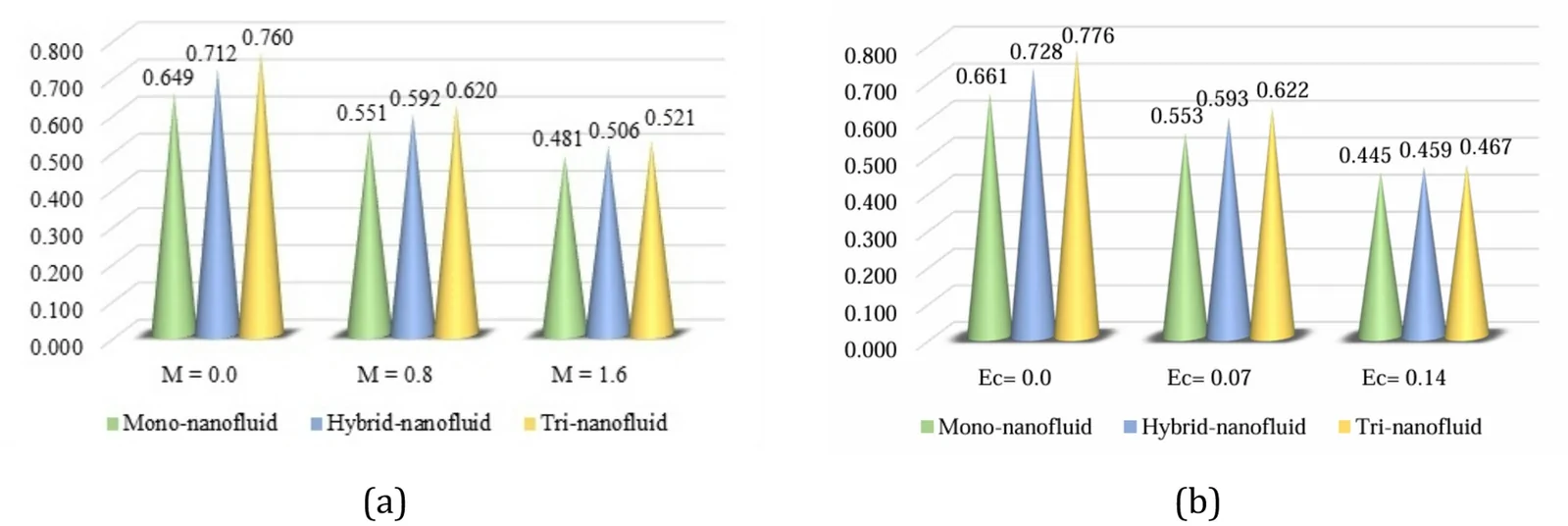
(**a**, **b**) Variation of $\:R{e}_{x}^{-\frac{1}{2}}\:Nu\:$ for mono, hybrid, and tri-nanofluids with different Hartmann number $\:\left(M\right)$ and Eckert number $\:\left(Ec\right)$, at fixed values of $\:A=0.2,\:Bn=0.2,\:Fr=0.2,R=0.1,{m}^{*}=0.1~\mathrm{a}\mathrm{n}\mathrm{d}~\:Pr=6.45\:$ considering thermal radiation and heat generation effects.

**Fig. 6:**
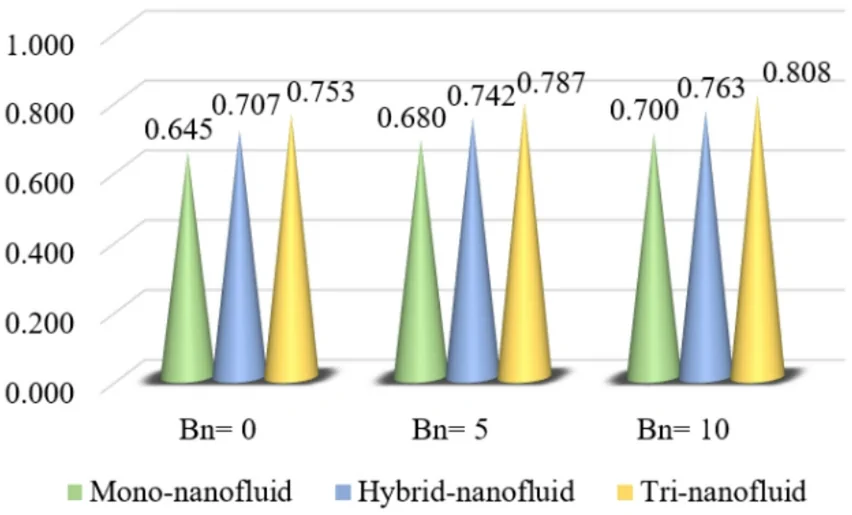
Variation of $\:R{e}_{x}^{-\frac{1}{2}}\:Nu\:$ for mono, hybrid, and tri-nanofluids with different Bingham number * (Bn*), at fixed values of $\:A=0.2,\:Da=0.1,\:Fr=0.2,R=0.1,{m}^{*}=0.1$, $\:Pr=6.45,Ec=0.05\:\:~\mathrm{a}\mathrm{n}\mathrm{d}\:~M=0.5\:$ considering thermal radiation and heat generation effects.

The wall heat transfer rate (Nusselt number) is an important quantity that provides fundamental information to design efficient and sustainable thermal systems. Therefore, its behavior under the variation of related physical parameters ($\:{m}^{*},n,\:\:R,M,Ec,\:Da$ and * Fr*) is studied. The Nusselt number is calculated versus parameters $\:R,\:M,\:Ec,Da,\:Fr$ and $\:{m}^{*}$. These computed values are displayed in Table 5. For a quick view of the behavior of$\:\left({Re}^{-\frac{1}{2}}\:Nu\right)$, bar graphs are plotted, which are given in Figs. 2a,b, 3, 4, 5, 6. The trend analysis is done for both mono-nano-Bingham fluid and di-nano-Bingham fluid $\:\left({Re}^{-\frac{1}{2}}\:Nu\right)$. The parameters $\:{m}^{*},\:n,\:R,\:M,\:Ec,Da,\:Fr$ determine the effects of thermal radiation, Joule heating, viscous dissipation, Darcy porous medium, and Forchheimer porous medium on the Nusselt number. Figure 2a,b, respectively, demonstrate the impact of variation in $\:{m}^{*}$, and * n* on the Nusselt number. It is evident from these Figures that the Nusselt number increases when $\:{m}^{*}$, and * n* are increased. Thus, the Bingham fluid with higher $\:{m}^{*}$, and * n* have more capability to transfer heat from the solid surface to the fluid. Similarly, the Nusselt number increases when $\:Pr\:$and * R* are increased, as shown in Fig. 3a,b. The impact of porous media (Darcy and Forchheimer) through * Da* and * Fr* is investigated, and related behaviors are displayed in Figs. 4a,b, which indicate a decrease in the Nusselt number versus * Da* and * Fr*. Moreover, Fig. 5a,b indicate that the Nusselt number decreases when the Hartmann number * M* and the Eckert number $\:Ec\:$are decreased. Similarly, the Bingham number * Bn* also has a decreasing effect on the Nusselt number as shown in Fig. 6. For thermal and cooling systems to be thermally efficient and sustainable, the working Bingham fluid should not be heat dissipative either because of Joule heating or viscous dissipation. However, thermal radiation causes a remarkable decrease in the temperature of the fluid due to the escape of heat energy via electromagnetic waves emitted by thermal radiation, and therefore, the wall heat transfer rate increases when the intensity of thermal radiation is increased. Moreover, the Bingham fluids with higher growth parameters are more efficient working fluids for thermal and cooling systems. Among the different types of nanofluids, this effect is most prominent in tri-nanofluids due to their higher thermal conductivity and stronger fluid–thermal interactions, which makes them more sensitive to parameter variations compared to mono- and di-nanofluids.

**Table 5:** The behavior of the Nusselt number for mono-nanofluid $\:Cu\:/SA$), di-nanofluid ($\:Cu-F{e}_{3}{O}_{4}/SA$), tri-nanofluid ($\:Cu-\:F{e}_{3}{O}_{4}-GO/SA$) under the change in related parameters.

*R*	M	* Ec*	Da	Fr	*N*	m*	* Bn*	Mono-nanofluid	Di-nanofluid	Tri-nanofluid
0.0	0.5	0.05	0.1	0.2	0.1	0.1	0.2	0.569897	0.621093	0.657683
0.2								0.597217	0.642269	0.674163
0.4								0.623436	0.663770	0.691996
0.1	0.0	0.05	0.1	0.2	0.1	0.1	0.2	0.649380	0.712290	0.759979
	0.8							0.551419	0.592238	0.620213
	1.6							0.480551	0.506035	0.521214
0.1	0.5	0.0	0.1	0.2	0.1	0.1	0.2	0.660926	0.727511	0.775793
		0.07						0.552802	0.593237	0.621646
		0.14						0.444678	0.458962	0.467499
0.1	0.5	0.05	0.0	0.2	0.1	0.1	0.2	0.595395	0.645615	0.681722
			1.5					0.458327	0.481473	0.494958
			3.0					0.362875	0.366620	0.364607
0.1	0.5	0.05	0.1	0	0.1	0.1	0.2	0.606438	0.657080	0.692421
				1				0.513665	0.552332	0.581669
				2				0.450898	0.480416	0.504618
0.1	0.5	0.05	0.1	0.2	0.0	0.1	0.2	0.472497	0.508516	0.533770
					0.1			0.583695	0.631601	0.665688
					0.2			0.682252	0.740700	0.782669
0.1	0.5	0.05	0.1	0.2	0.1	0	0.2	0.583119	0.631102	0.665237
						5		0.616659	0.661620	0.693309
						10		0.611059	0.653088	0.682144
0.1	0.5	0.01	0.1	0.2	0.1	0.2	0	0.644542	0.707434	0.752900
							5	0.679835	0.742069	0.787159
							10	0.700278	0.763030	0.808368

### The Predictions on the trend of the skin friction coefficient $\:\sqrt{\boldsymbol{R}\boldsymbol{e}}\:{\boldsymbol{C}}_{\boldsymbol{f}}$

**Fig. 7:**
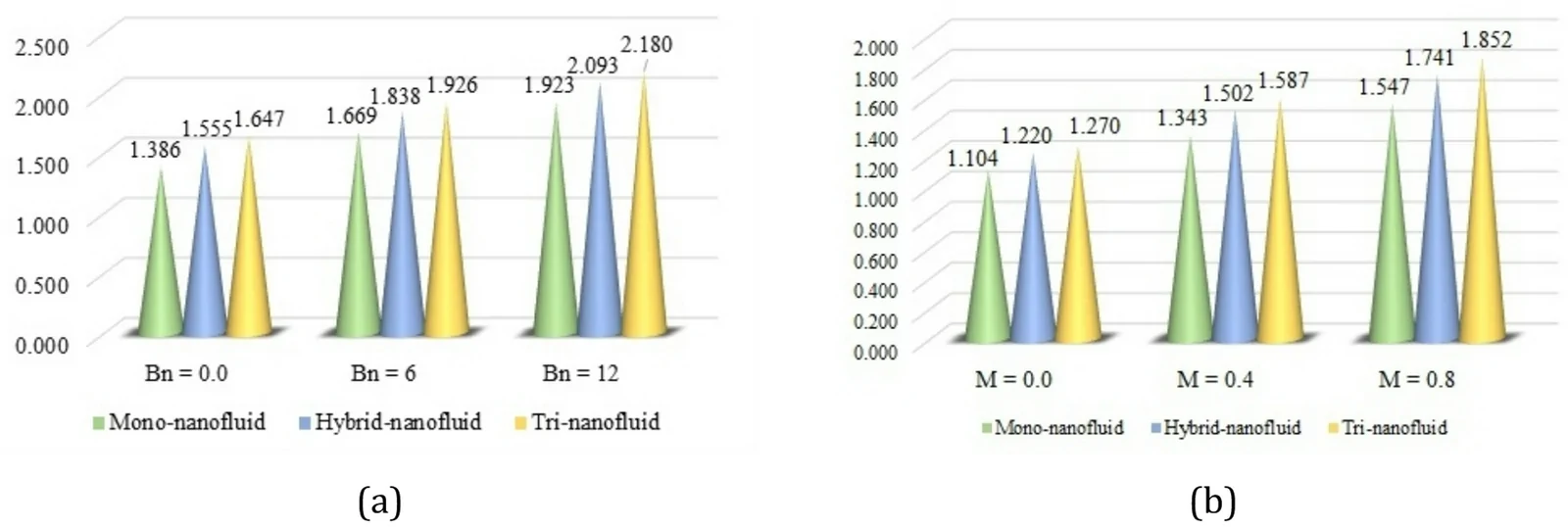
(**a**, **b**) Variation of $\:R{e}_{x}^{-\frac{1}{2}}\:{C}_{f}\:$ for mono, hybrid, and tri-nanofluids with different Darcy numbers $\:\left(Da\right)$ and Bingham numbers $\:\left(Bn\right)$, at fixed values of $\:A=0.2,\:M=0.2,{m}^{*}=0.1\:$ and $\:\:Fr=0.1\:$considering thermal radiation and heat generation effects.

**Fig. 8:**
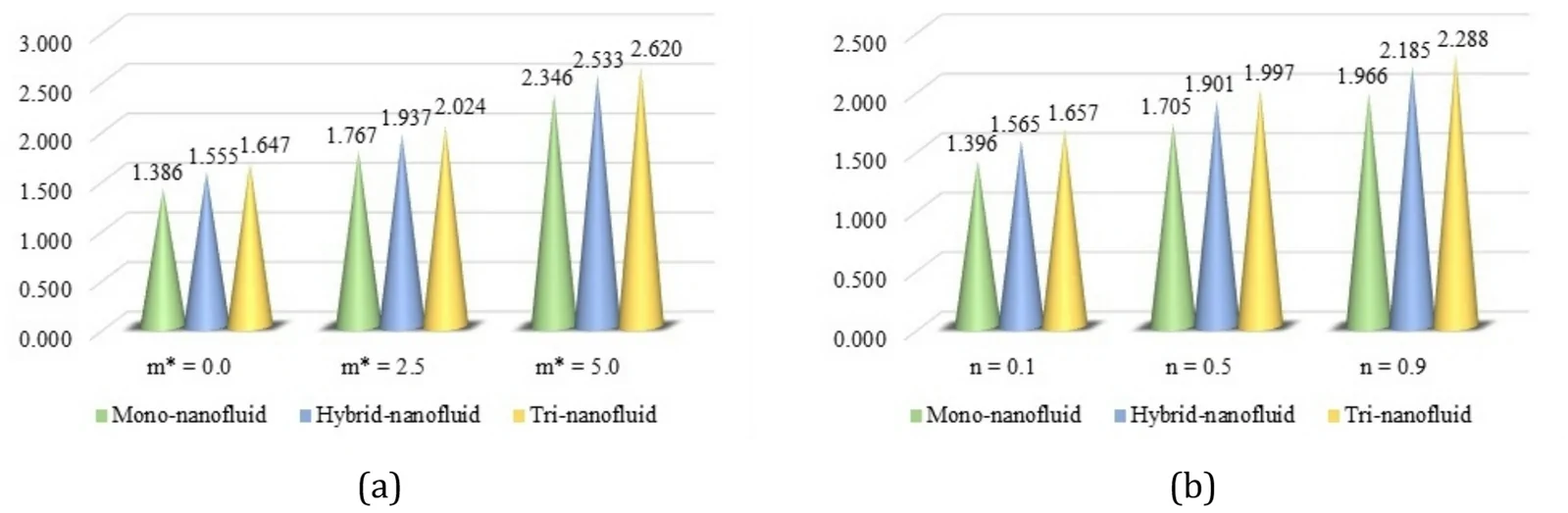
(**a**, **b**): Variation of $\:R{e}_{x}^{-\frac{1}{2}}\:{C}_{f}\:$ for mono, hybrid, and tri-nanofluids with different growth parameter $\:\left({m}^{*}\right)$ and power-law index $\:\left(n\right)$, at fixed values of $\:A=0.2,\:M=0.2,Da=0.1,Bn=0.2\:$ and $\:\:Fr=0.1\:$ considering thermal radiation and heat generation effects.

**Fig. 9:**
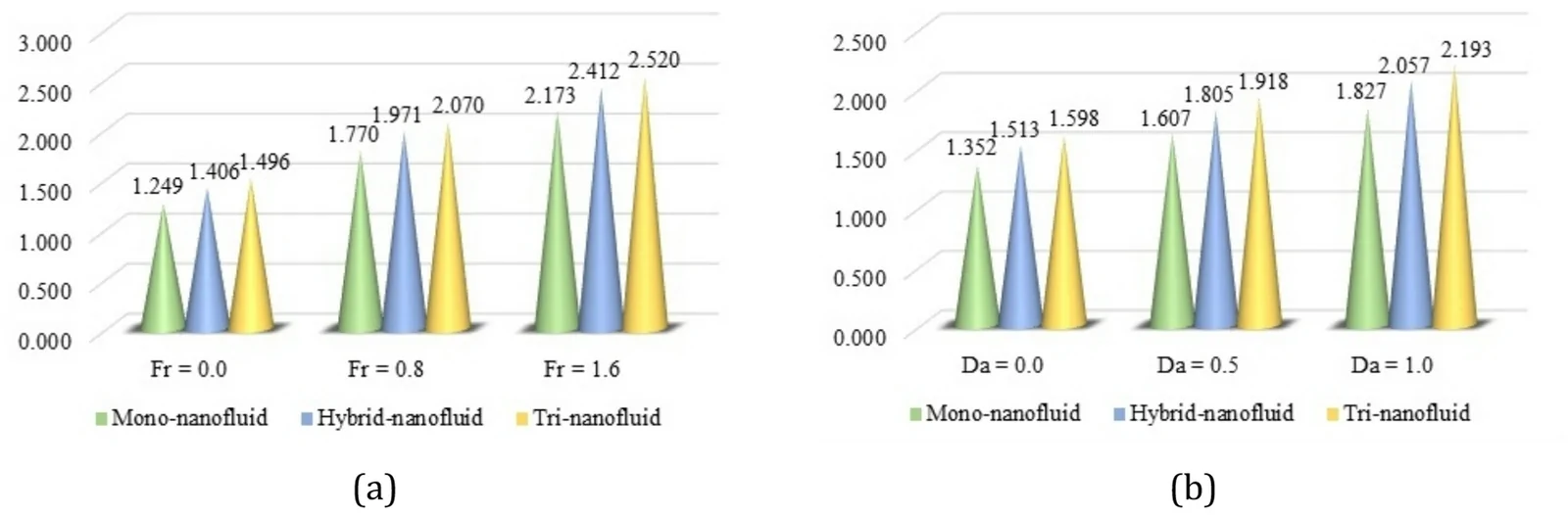
(**a**, **b**): Variation of $\:R{e}_{x}^{-\frac{1}{2}}\:{C}_{f}\:$ for mono, hybrid, and tri-nanofluids with different Forchheimer porous medium $\:\left(Fr\right)$ and Hartmann number $\:\:\left(M\right)$, at fixed values of $\:A=0.2,\:Bn=0.2,Da=0.1\:$ and $\:\:{m}^{*}=0.1\:$considering thermal radiation and heat generation effects.

The skin friction coefficient $\:\left(\sqrt{Re}\:{C}_{f}\right)$ is an important physical quantity that provides important predictions regarding fluid flows and drag on the solid surface during fluid-solid interactions. The parameter affecting $\:\sqrt{Re}\:{C}_{f}$ in this study are $\:M,\:Bn,\:Da,\:Fr$ and $\:{m}^{*}$. Therefore, $\:\sqrt{Re}\:{C}_{f}$ is computed versus variation in these parameters, and these computed values are given in Table 6. This Table shows that the Bingham fluid with $\:Cu,\:F{e}_{3}{O}_{4}$ and * GO* exerts the highest stear stress in comparison with * Cu* and * Cu* and $\:F{e}_{3}{O}_{4}$. Moreover, magnetic force and porous medium forces are responsible for an increase in wall shear stress. In addition to this, a remarkable increase in wall shear stress is noticed when the Bingham number is increased. Thus, in fluid-solid interaction, the Bingham fluid with higher yield stress exerts high shear stress on the surface of the solid. Further, wall shear stress increases with an increase in the growth parameter. Table 6; Figs. 6a,b, 7, 8 and 9a,b show that increasing the Bingham number $\:\left(Bn\right)$, growth parameter $\:\left({m}^{*}\right)$, and power index $\:\left(n\right)$ leads to a noticeable rise in skin friction, which significantly increases shear stress on the surface. Collectively, these effects raise the surface stress to levels that can lead to erosion or structural damage. This effect becomes even more critical in tri-nanofluids as compared to mono and di-nanofluids. These observations can be confirmed from Fig. 6a,b, 7a,b, and 8a,b. The parameters * Da* and * Fr*, are called the Darcy-porous medium parameter and Forchheimer porous medium parameter, and determine the influence of porous medium force on fluid flows. The effects are given in Fig. 9a,b, respectively. These Figures indicate that by increasing * Da* and $\:Fr\:$ on the velocity of the flow decreases. This further indicates a decrease in the momentum boundary layer thickness. Thus, the porous medium plays a crucial role in controlling the thickness of the viscous region. Moreover, viscous mono nanofluid experiences the highest retardation by the porous medium. It is also noted from Figs. 6a,b, 7, 8 and 9a,b that tri-nanofluid exerts the highest wall shear stress in comparison with mono-, and di-nanofluids.

**Table 6:** The behavior of the skin friction coefficient of nanofluids (mono-nanofluid $\:Cu\:/SA$) di-nanofluid ($\:Cu-F{e}_{3}{O}_{4}/SA$), tri-nanofluid ($\:Cu-\:F{e}_{3}{O}_{4}-GO/SA$).

M	* Bn*	Da	Fr	*n*	m*	* mono*	* di*	* tri*
0.0	0.2	0.1	0.1	0.1	0.1	1.103959	1.220335	1.269558
0.4						1.342538	1.502276	1.586504
0.8						1.546649	1.741378	1.852453
0.2	0	0.1	0.1	0.1	0.1	1.386109	1.555316	1.646987
	6					1.669441	1.837682	1.926062
	12					1.922685	2.092616	2.179656
0.2	0.2	0.0	0.1	0.1	0.1	1.351801	1.512734	1.597904
		0.5				1.606700	1.804810	1.917987
		1.0				1.827379	2.056886	2.192914
0.2	0.2	0.1	0.0	0.1	0.1	1.249097	1.406195	1.495936
			0.8			1.769839	1.971280	2.069685
			1.6			2.173433	2.411775	2.520191
0.2	0.2	0.1	0.1	0.1	0.1	1.396237	1.565332	1.656841
				0.5		1.704979	1.900525	1.997372
				0.9		1.965978	2.185025	2.287864
0.2	0.2	0.1	0.1	0.1	0.0	1.386109	1.555316	1.646987
					2.5	1.767378	1.937305	2.023597
					5.0	2.345816	2.533366	2.620181

### Temperature distribution versus related parameters

**Fig. 10:**
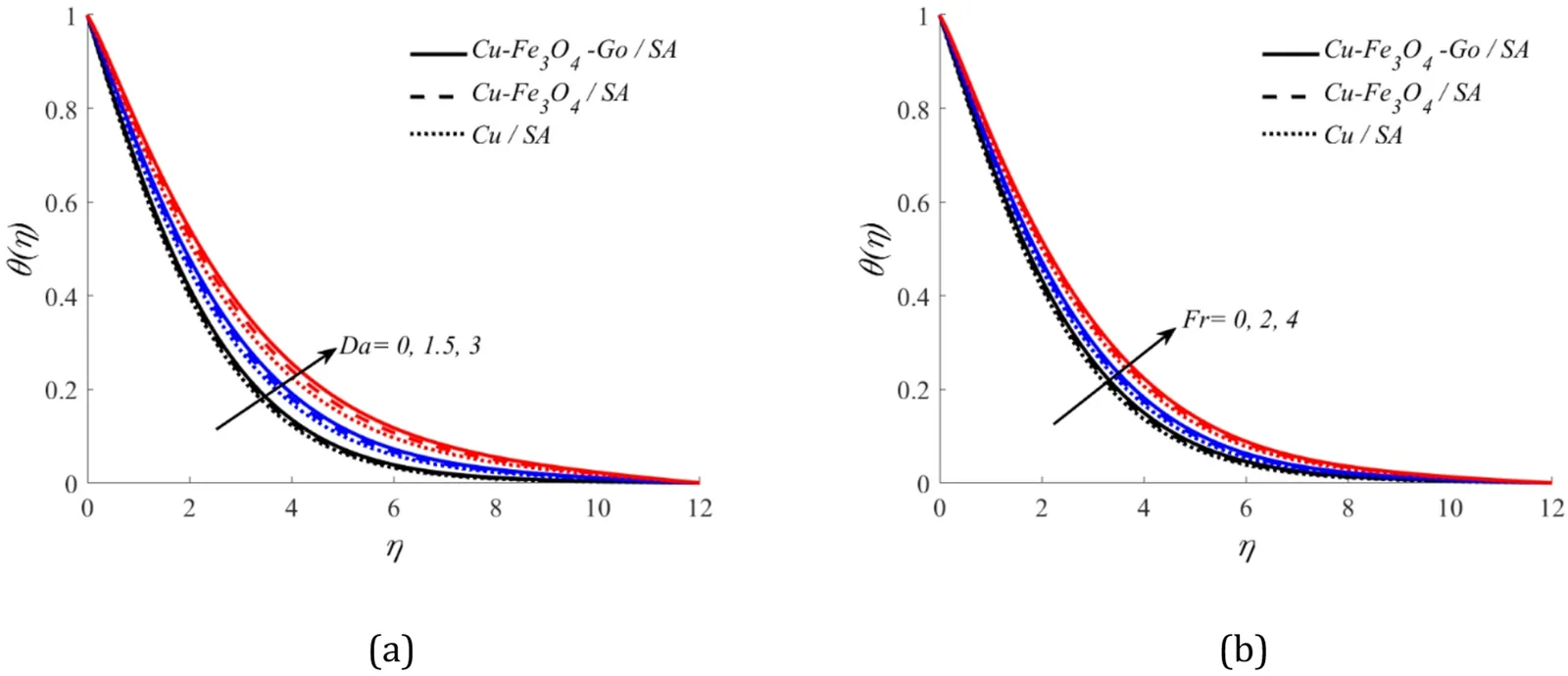
(**a**, **b**): Influence of Darcy number * Da* and Forchheimer porous medium $\:Fr\:$ on $\:\theta\:\left(\eta\:\right)$ when $\:A=0.2,\:Bn=0.2,\:R=0.1,Pr=6.45$,$\:Ec=0.05,M=0.5,\:$ and $\:{m}^{*}=0.1$.

**Fig. 11:**
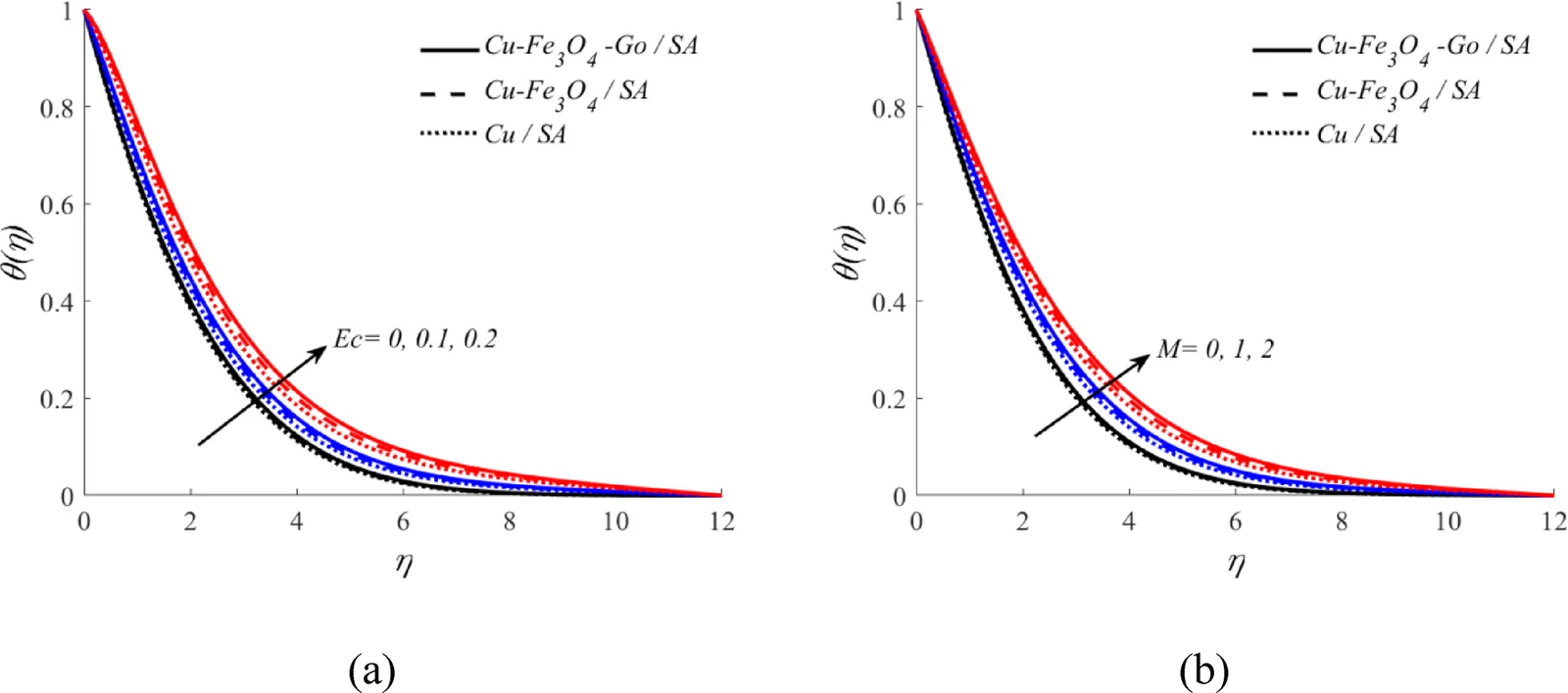
**(a**, **b**): Influence of Eckert number * Ec* and Hartmann number $\:M\:$ on $\:\theta\:\left(\eta\:\right)$ when $\:A=0.2,\:Bn=0.2,\:R=0.1,Pr=6.45$,$\:Da=0.1,Fr=0.2,\:$ and $\:\:{m}^{*}=0.1$.

**Fig. 12:**
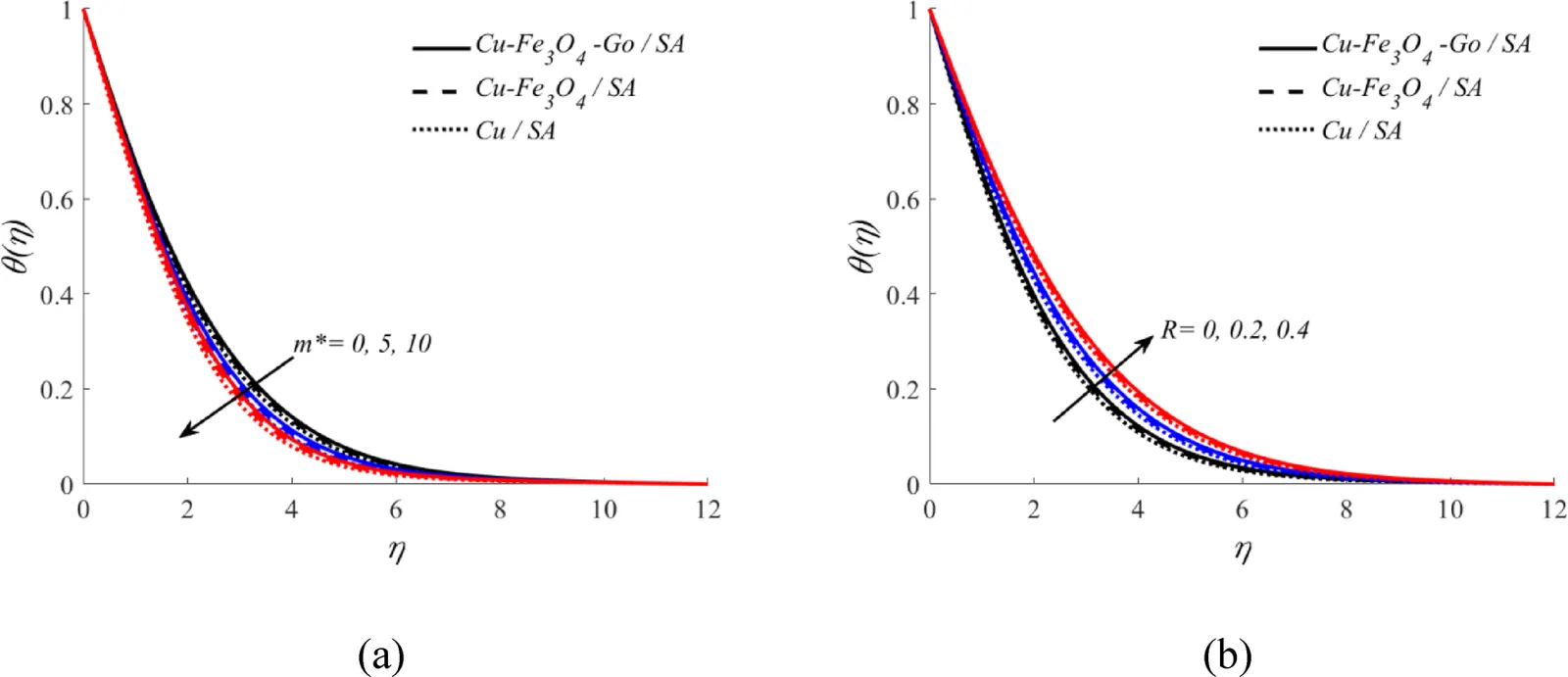
(**a**, **b**) Influence of growth parameter $\:\:{m}^{*}$ and radiation parameter * R* on $\:\theta\:\left(\eta\:\right)$ when$\:A=0.2,\:Bn=0.2,Fr=0.2,Pr=6.45$,$\:Ec=0.05,M=0.5,\:$ and $\:\:Da=0.1$.

The parameters $\:Da,\:Fr,\:Ec$ and $\:M\:$ affecting temperature distribution and therefore, the impact on temperature field in mono, di-, and tri nano-Bingham fluids are examined in Fig. 10a,b, 11 and 12a,b. In Fig. 10a, temperature profiles show an increasing trend, and $\:Da\:$ is increased. This increasing trend is because of the dissipation of heat resulted in because of friction force by Darcy porous medium. This increasing trend in temperature distribution is observed in mono-, di-, and tri nano-Bingham fluid. Moreover, thermal boundary layer thickness (TBLT) increases because of the accumulation of heat dissipated due to the friction force of the Darcy porous medium. A similar trend in temperature distribution versus Forchheimer porous medium parameter (* Fr*) is observed (see Fig. 10b). The Eckert and Hartmann numbers ($\:Ec\:$ and $\:\:M$) are coefficients of terms in the energy equation arising due to the consideration of viscous dissipation and Joule heating. Both phenomena produce heat, which accumulates in the fluid, and consequently, the temperature of the fluid has shown an increasing trend. This trend is observed in three types of nanofluids considered here in this study (see Fig. 11a,b). Figure 11a,b indicates that Joule heating and viscous dissipation effects in tri nano-Bingham fluid are stronger than those of the other di and mono nano-Bingham fluids. Figure 12a examines the behavior of the growth parameter ($\:{m}^{*}$) on temperature distribution for the three types of nanofluids. The influence of thermal radiation on temperature distribution is given in Fig. 12b which shows an increase in temperature when * R* is increased. It is important to note that an increase in * R* indicates decrease in emission of thermal radiations and therefore, escaping of heat energy through electromagnetic waves decreases and temperature of fluid increases.

### Velocity distribution versus related parameters

**Fig. 13:**
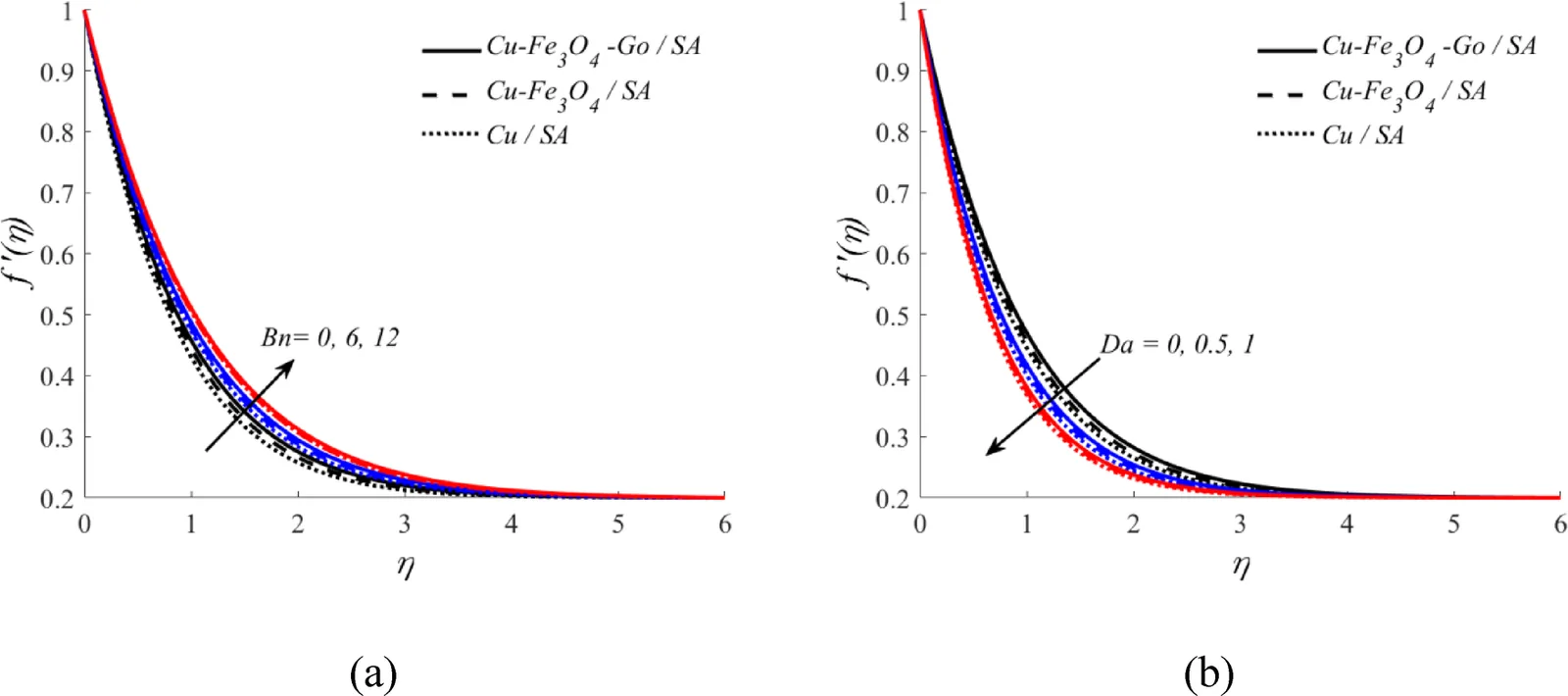
(**a**, **b**): Influence of Bingham number * Bn* and Darcy number $\:Da\:$ on $\:f^{\prime\:}\left(\eta\:\right)$ when $\:A=0.2,\:M=0.2,{m}^{*}=0.1\:$ and $\:\:Fr=0.1$.

**Fig. 14:**
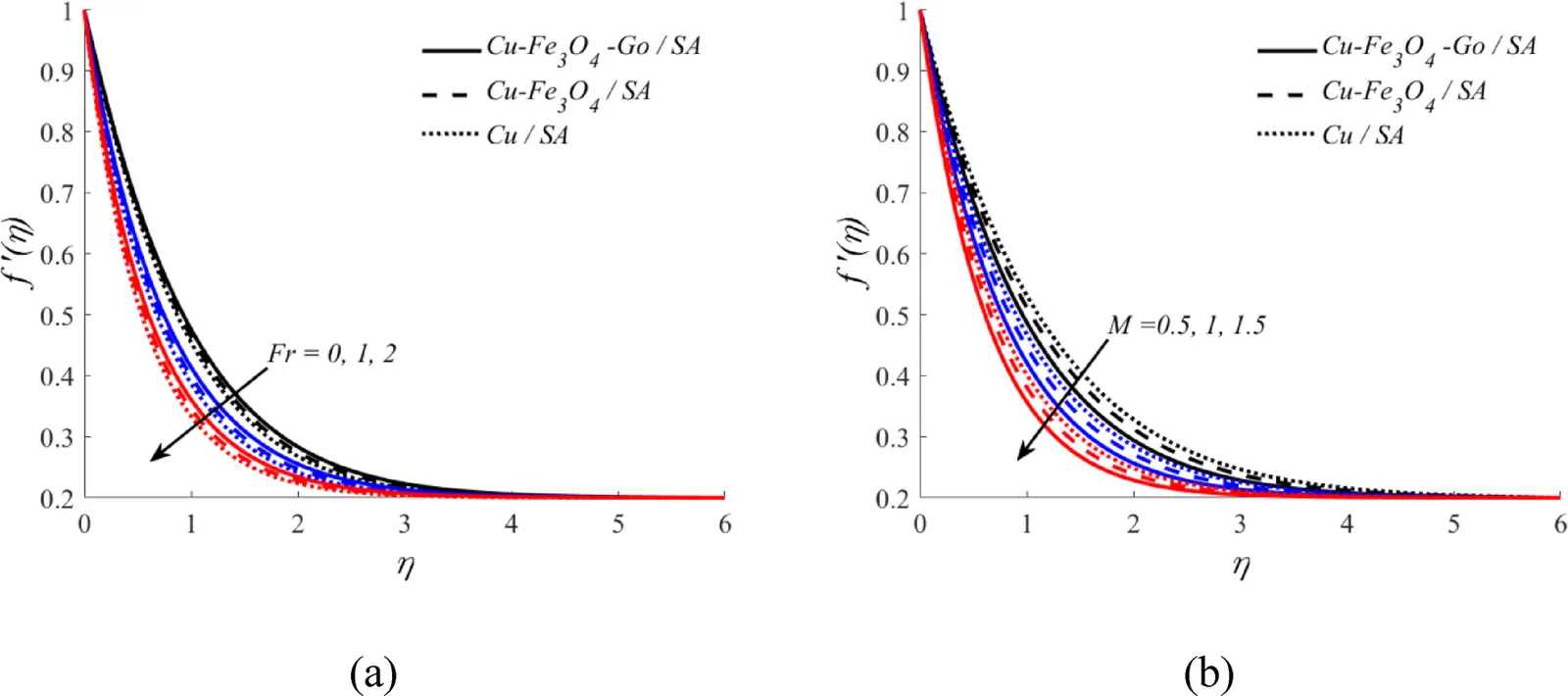
(**a**, **b**) Influence of Forchheimer number * Fr* and Hartmann number * M* on $\:f^{\prime\:}\left(\eta\:\right)$ when $\:A=0.2,\:Bn=0.2,\:{m}^{*}=0.1\:$ and * Da=0.1*.

Fluid flow simulations are performed to analyze the impact of key parameters determining different flow conditions. The behavior of yield stress (viscoplasticity), Darcy porous medium, Forchheimer porous medium, magnetic field, and regularization can be studied through variation of parameters $\:Bn,\:Da,\:Fr,\:M$ and $\:{m}^{*}$, and related simulations are given in Figs. 13a,b and 14a,b. The parameter called the Bingham number $\:\left(Bn\right)$ determines the impact of viscoplasticity on the velocity of $\:Cu+F{e}_{3}{O}_{4}+Go+SA$, $\:Cu+F{e}_{3}{O}_{4}+SA$, * Cu+SA* in Fig. 13a. This Figure shows that the motion of fluid accelerates if the fluid over a wedge has a higher yield stress. Hence, Newtonian fluid diffuses wall momentum (momentum of the wedge) more slowly than the Bingham fluid. It is also observed from Fig. 13b that $\:Cu+F{e}_{3}{O}_{4}+Go+SA$ absorbs wall momentum faster than the other considered fluids. Figures 13b and 14a study the influence of Darcy and Forchheimer porous media on the velocity of the considered fluid flows. In both Darcy and Forchheimer porous media, the velocity of the fluid decreases because the porous media in the fluid offer resistance to the fluid flow, and is crucial in controlling the momentum boundary layer thickness. This observation is evident from Fig. 14b that the Lorentz force is also a resistive force and slows down the motion of fluids, and eventually the thickness of the boundary layer region decreases, as shown in Fig. 14b.

## Conclusions

Boundary problems associated with the simultaneous transfer of heat and flow of the Brigham fluid over a wedge with a heat surface placed in a Darcy and a Forchheimer porous media are solved numerically. The computed numerical solutions are further utilized for investigating the influence of nanoparticles in enhancing the thermal performance of fluid containing $\:Cu,F{e}_{3}{O}_{4}$ and * GO*.The Skin friction coefficient, the Nusselt number, temperature, and velocity fields are studied, and the following key conclusions are drawn from numerical simulations.

- For thermal and cooling systems to be thermally efficient and sustainable, the working Bingham fluid should not be heat dissipative, either because of Joule heating or viscous dissipation.
- Among the different types of nanofluids, the most prominent is the case of tri-nanofluids due to their higher thermal conductivity and stronger fluid–thermal interactions, which makes them more sensitive to parameter variations compared to mono- and di-nanofluids.
- The porous medium causes dissipation due to the resistive force experienced by the fluid flow. This dissipated heat adds to the fluid and hence its temperature. This increase in temperature results in a decrease in the Nusselt number.
- The resistive forces by the magnetic field and porous medium cause an increase in the wall Shear stress. Hence, fluid-solid interaction in a porous medium in the presence of a magnetic force results in a high drag on the surface of the wedge.
- An increase in thermal radiation intensity leads to an increase in the Nusselt number. Thus, thermally radiative fluids are thermally more efficient in transporting heat.
- The numerical simulations have given predictions that the skin friction coefficient increases when the Bingham number is increased. Hence, the viscoplastic fluids with higher yield stress exert higher drag on the solid surface to which such fluid interacts. Moreover, the viscoplastic fluid (the Bingham fluid) exerts higher drag on the surface in comparison with Newtonian fluids.
- The porous medium can be used for controlling the momentum boundary layer thickness. However, the presence of porous medium in the fluid causes an increase in thermal boundary layer thickness (TBLT). Similarly, Joule heating increases the TBLT.

## Data Availability

All data generated or analysed during this study are included in this published article.
